# Supplementary figures and images for: Integrative and quantitative view of the CtrA regulatory network in a stalked budding bacterium
Source: PLoS Genet. 2020 Apr 23;16(4):e1008724. doi: 10.1371/journal.pgen.1008724 (PMC7200025; doi:10.1371/journal.pgen.1008724)

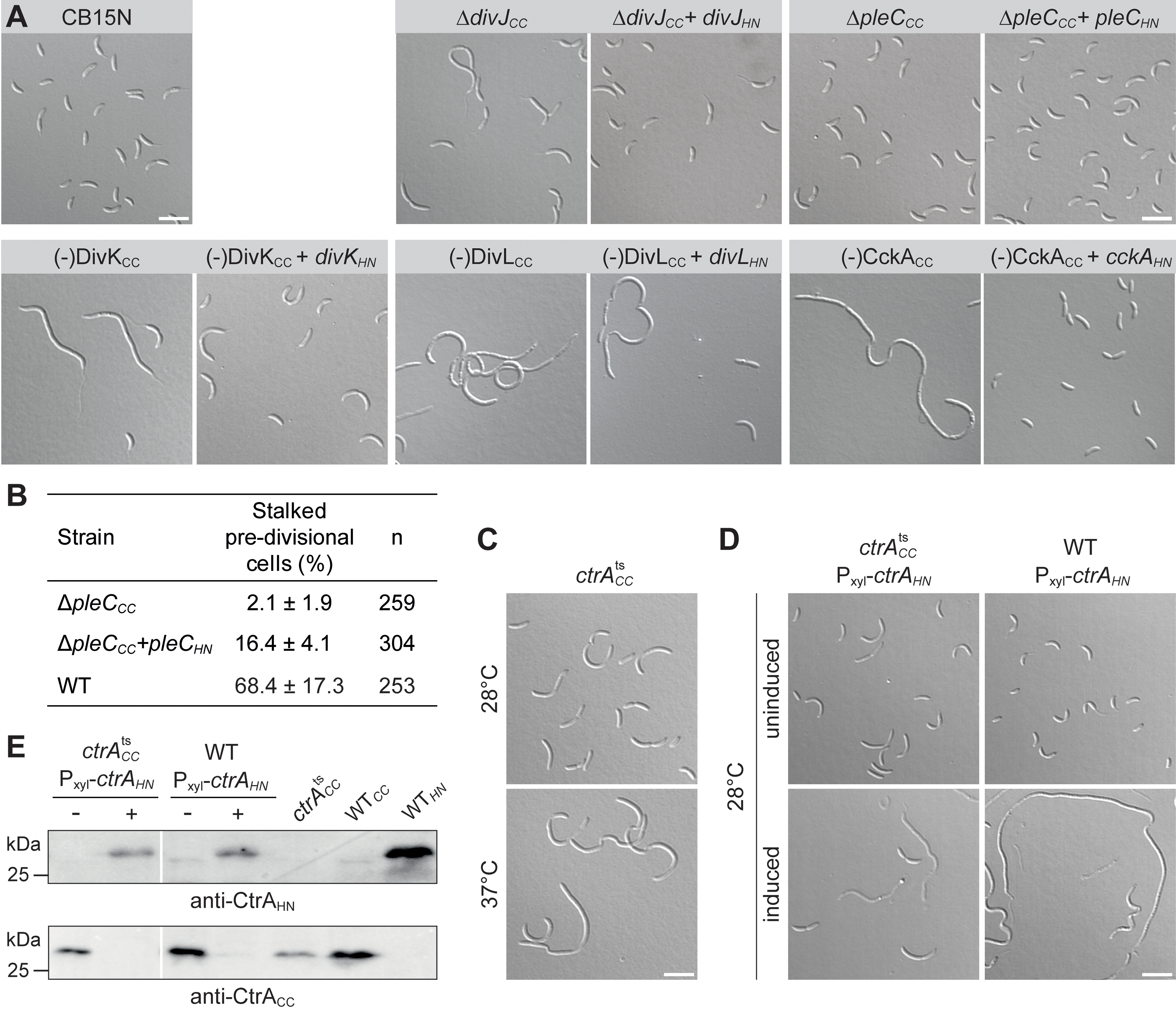

Supplement: S1 Fig — (A) DIC images of C. crescentus mutants with in-frame deletions in divJCC (YB3202) or pleCCC (UJ506) or temperature-sensitive mutations in divKCC (LS3570), divLCC (KR635) or cckACC (CckATS1) expressing divJHN (OL133), pleCHN (OL135), divKHN (OL137), divLHN (MvT81) or cckAHN (OL179), respectively, from a xylose-inducible promoter (under restrictive conditions). C. crescentus wild-type (CB15N) cells are shown for comparison. (B) Quantification of the fraction of stalked cells in cultures of a ΔpleCCC mutant carrying a xylose-inducible copy of pleCHN (OL135) in the absence or presence of inducer. (C) C. crescentus strain carrying a temperature-sensitive variant of CtrACC (LS2195) at the restrictive (37°C) and permissive (28°C) temperature. (D) Dominant negative effect of CtrAHN expressed from a xylose-inducible promoter (Pxyl) in cells producing either a temperature-sensitive (OL128; at the permissive temperature) or the wild-type (OL130) form of CtrACC. (E) Immunoblots showing the degradation of CtrACC upon induction of CtrAHN in C. crescentus. C. crescentus strains carrying a temperature-sensitive (OL128) or wild-type (OL130) allele of ctrACC and an ectopic copy of ctrAHN under the control of a xylose-inducible promoter were grown in the absence (-) or presence (+) of xylose and analyzed with anti-CtrAHN and anti-CtrACC antibodies. Cells of the C. crescentus ctrAts (LS2195) and wild-type (WTCC; CB15N) strains and the H. neptunium wild-type strain (WTHN; LE670) were analyzed as controls. All cultures analyzed in panels A-E were grown at 28°C, unless stated otherwise. Cells were withdrawn from exponential cultures after depletion and/or induction of the respective proteins for 24 h. Scale bars: 5 μm. (TIF) [file pgen.1008724.s001.tif]

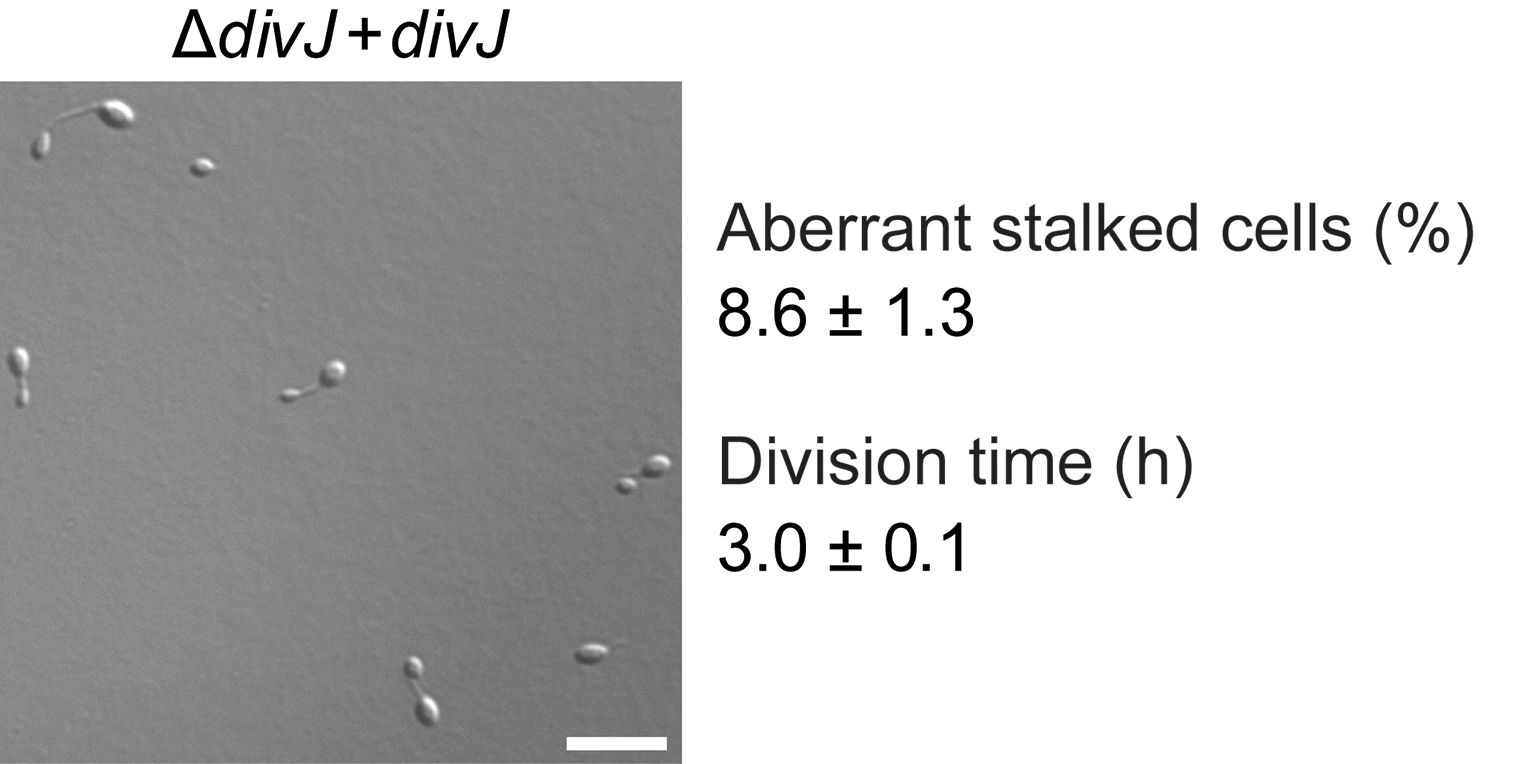

Supplement: S2 Fig — . An H. neptunium ΔdivJHN mutant carrying an ectopic copy of divJHN under the control of a copper-inducible promoter (OL123) was grown for 24 h in copper-containing medium and subjected to DIC microscopy. The percentage of stalked cells in the culture and the division time are shown on the right. Scale bar: 5 μm. (TIF) [file pgen.1008724.s002.tif]

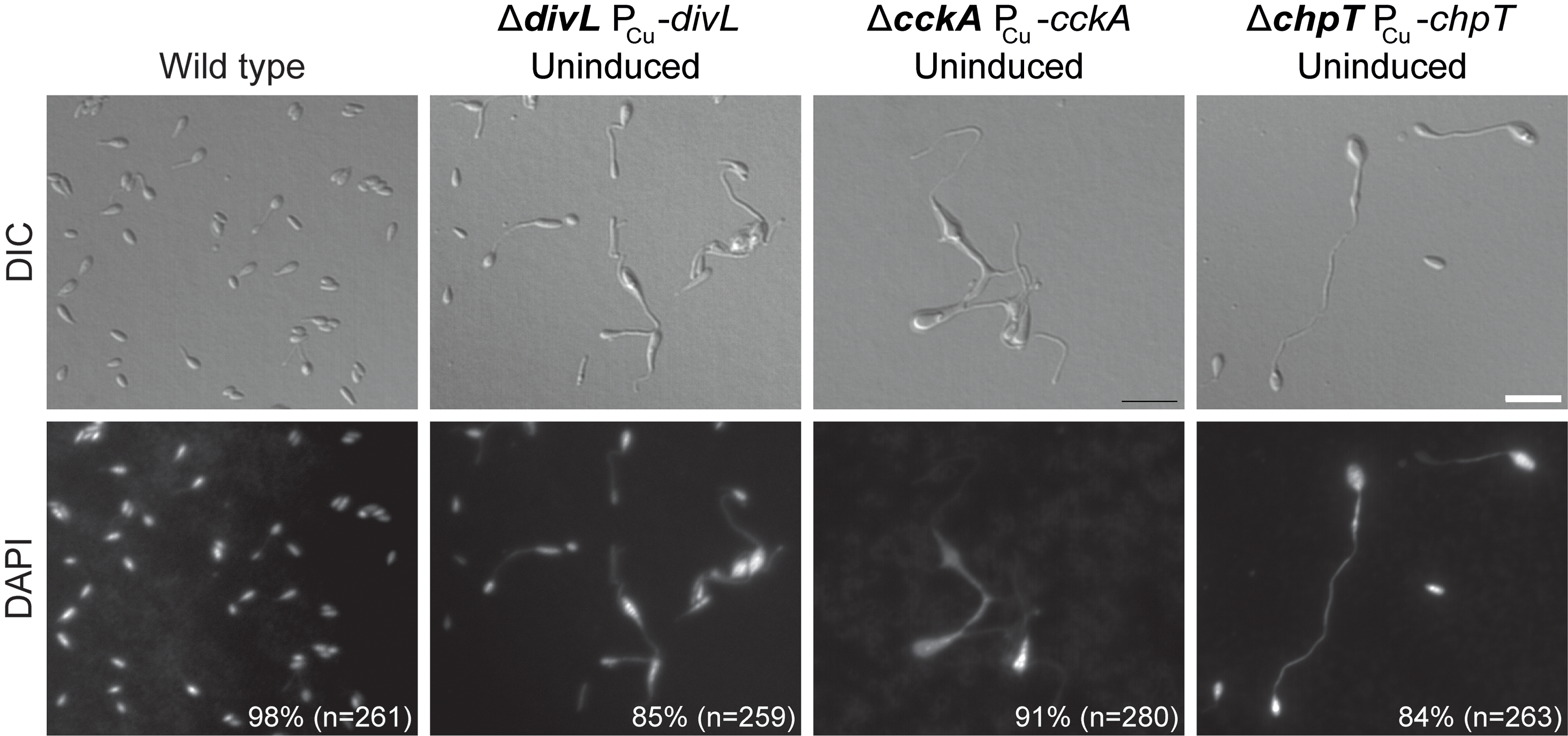

Supplement: S3 Fig — H. neptunium strains carrying conditional alleles of divLHN (OL177), cckAHN (OL161) or chpTHN (OL152) were grown for 24 h in the absence of inducer. Chromosomal DNA was stained with DAPI prior to imaging. Wild-type cells are shown for comparison. Scale bar: 5 μm. The percentage of cell bodies that show a DAPI signal is given in the bottom right corner of each fluorescence image. (TIF) [file pgen.1008724.s003.tif]

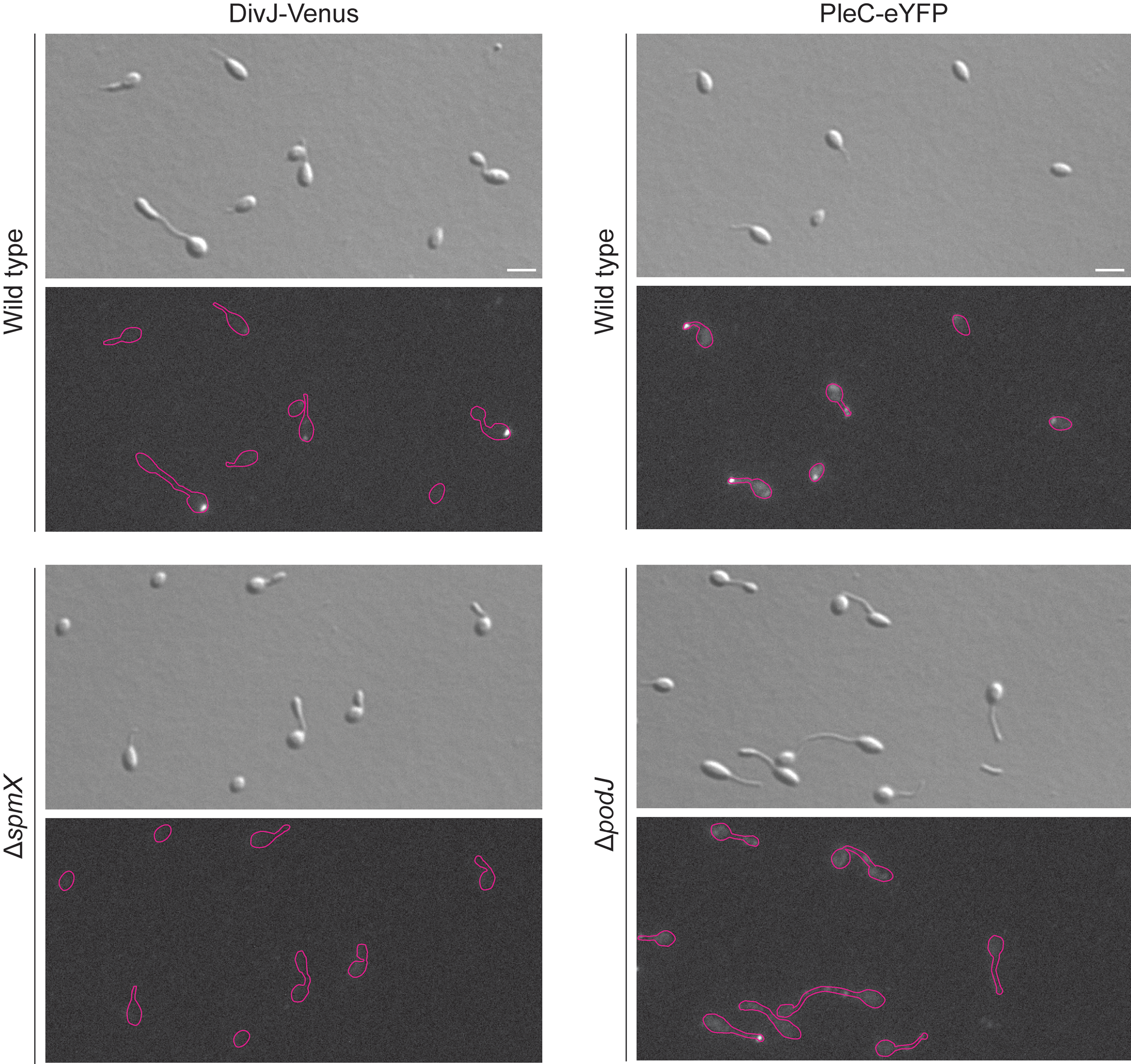

Supplement: S4 Fig — DivJ-Venus does not condense into distinct foci in cells lacking SpmX (OL36), whereas it shows the typical polar localization in the wild-type background (OL146). Similarly, PleC-eYFP foci are observed only sporadically in cells lacking PodJ (OL166), whereas they form normally in the wild-type background (OL151). Scale bars: 5 μm. (TIF) [file pgen.1008724.s004.tif]

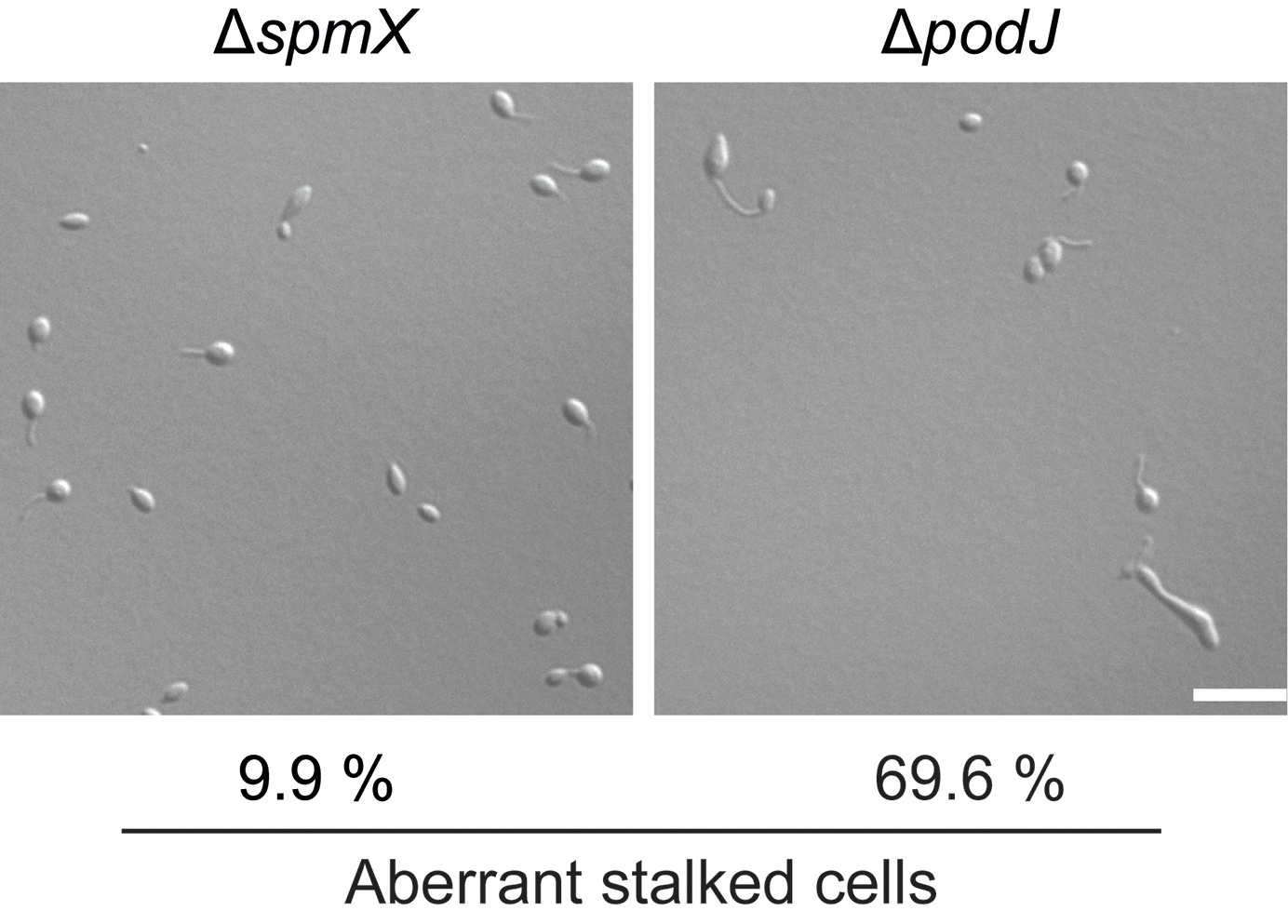

Supplement: S5 Fig — Shown are DIC images of H. neptunium ΔspmX (OL34) and ΔpodJ (OL35) cells. A quantification of the proportion of stalked cells with aberrant morphologies is given below the images. Scale bar: 5 μm. (TIF) [file pgen.1008724.s005.tif]

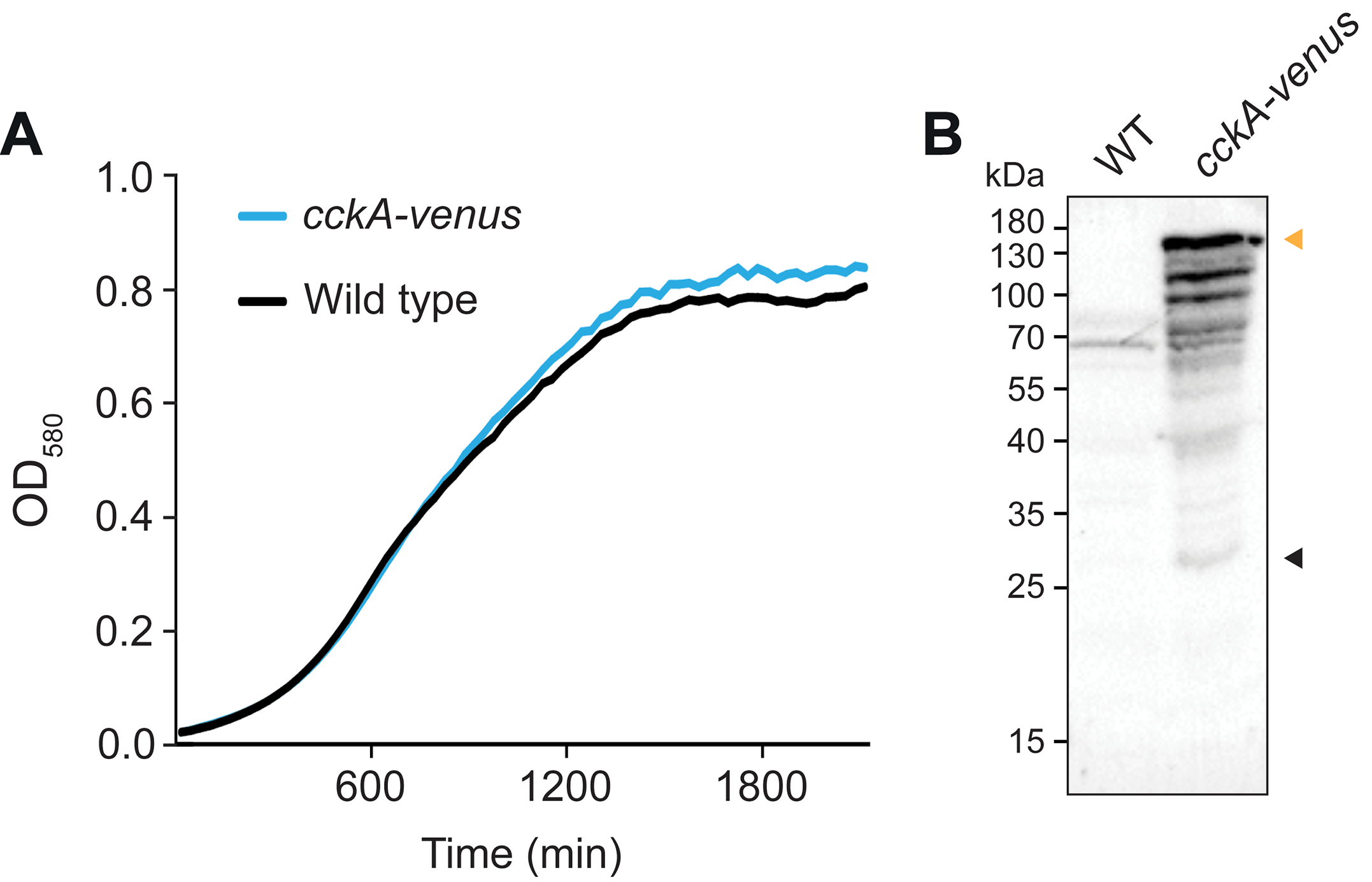

Supplement: S6 Fig — (A) Growth of an H. neptunium strain expressing cckA-venus in place of the native cckA gene (OL2). The growth of wild-type (LE760) cells is shown for comparison. Data represent the average of five independent experiments. (B) Immunoblot showing the accumulation of CckA-Venus. Samples of the strains analyzed in (A) were probed with anti-GFP antibodies. The full-length CckA-Venus fusion is indicated by an orange arrowhead. Cleaved Venus is indicated by a black arrowhead. (TIF) [file pgen.1008724.s006.tif]

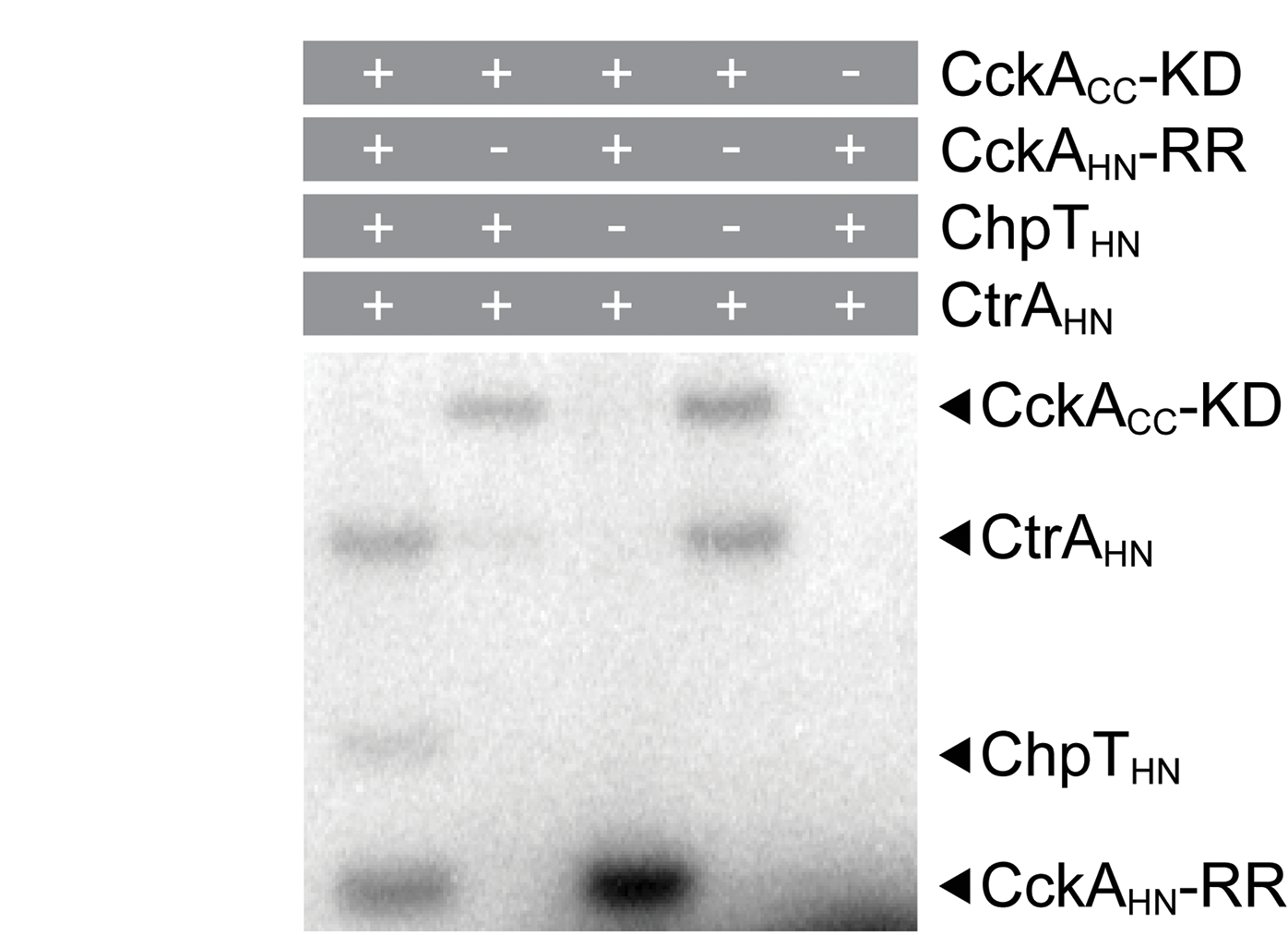

Supplement: S7 Fig — CckA-KDCC was autophosphorylated for 45 min at 30°C. Subsequently, the indicated proteins (marked with pluses) were combined and incubated for 5 min at 30°C. After termination of the reactions by addition of SDS sample buffer, proteins were separated by SDS-PAGE and radioactivity was detected by phosphor imaging. (TIF) [file pgen.1008724.s007.tif]

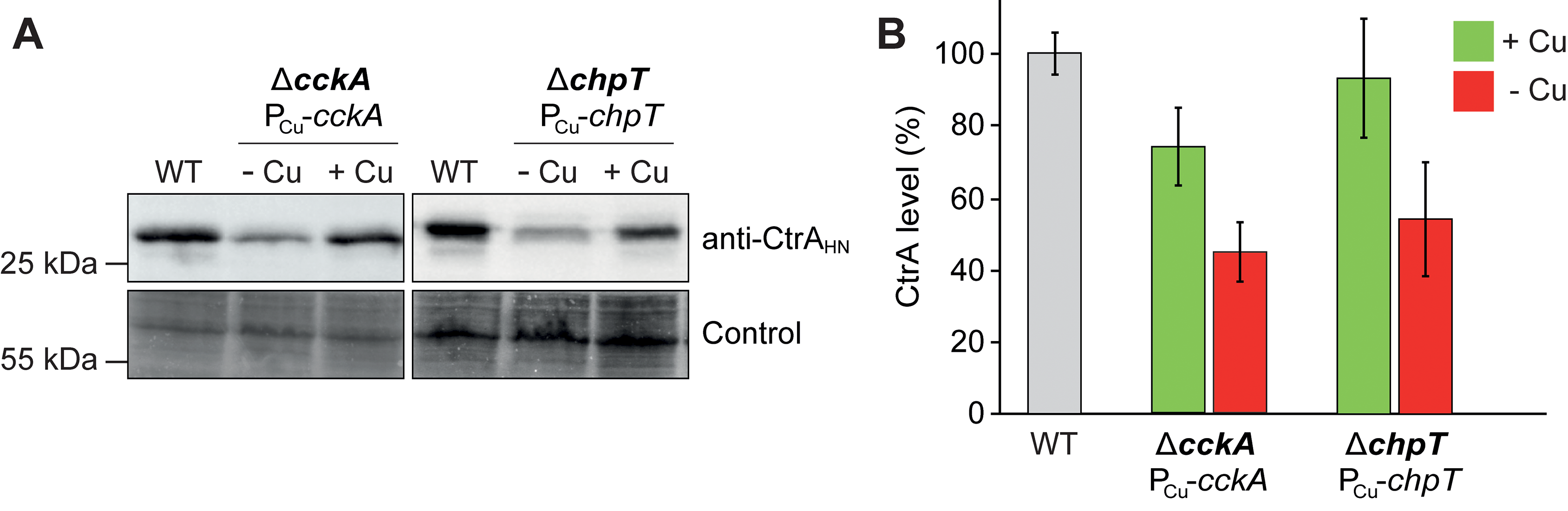

Supplement: S8 Fig — (A) Immunoblot showing the levels of CtrA after depletion of CckA or ChpT. Conditional H. neptunium mutants carrying copper-inducible copies of cckA (OL161) or chpT (OL152) were cultivated for 24 h in the absence of inducer and probed with anti-CtrAHN antibodies. Wild-type cells were analyzed for comparison. A representative section of the membrane stained with Amido black is shown as a loading control. (B) Quantification of the levels of CtrA after depletion of CckA or ChpT. The conditional cckA and chpT mutants analyzed in (A) were grown for 24 h in the presence (+ Cu) and absence (- Cu) of inducer and subjected to immunoblot analysis with anti-CtrAHN antibodies. The signals were quantified and normalized to the signal obtained for wild-type control cells. Data represent the average of three biological replicates, each of which was analyzed in triplicate. Error bars indicate the standard deviation. (TIF) [file pgen.1008724.s008.tif]

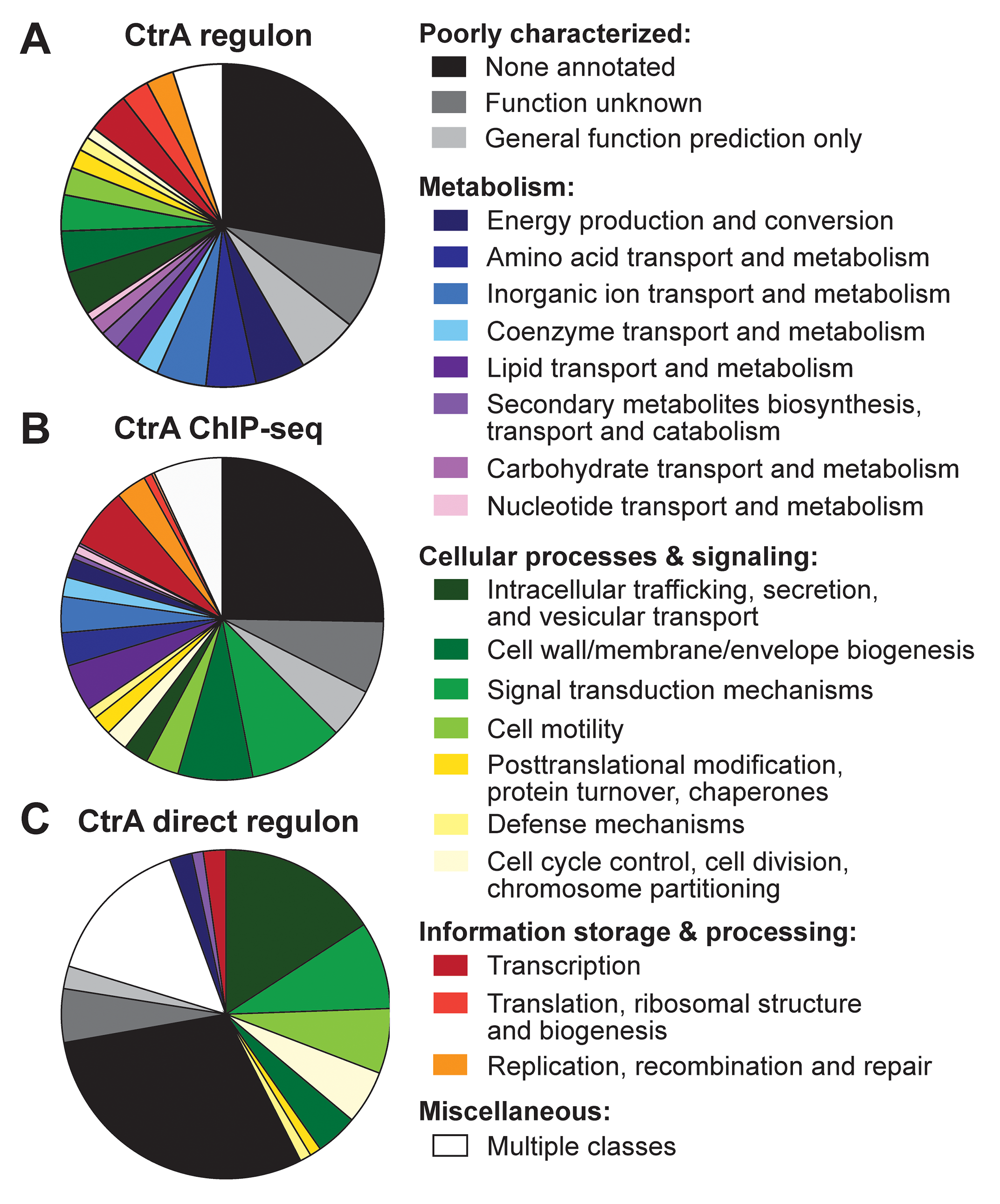

Supplement: S9 Fig — (A) Overview of the proportion of different COG categories among the 381 genes that are differentially expressed upon depletion of CckA and ChpT (see Fig 8A). Only genes with an RPKM value of >25, a p-value of <0.05 and a log2-fold change in expression of >2 were taken in account. (B) Overview of the proportion of different COG categories among the 285 genes contained in the transcriptional units that are immediately adjacent to the 222 CtrA binding sites identified in this study (see Fig 8B). (C) Venn diagram showing the number of genes (single or in an operon) bound by CtrA (in blue) and the number of genes present in the entire CtrA regulon (in red). The intersection of these two gene sets defines the direct CtrA regulon and comprises 94 genes. (C) Overview of the abundance of different COG categories among the 94 genes contained in the direct CtrA regulon (see Fig 8D). (TIF) [file pgen.1008724.s009.tif]

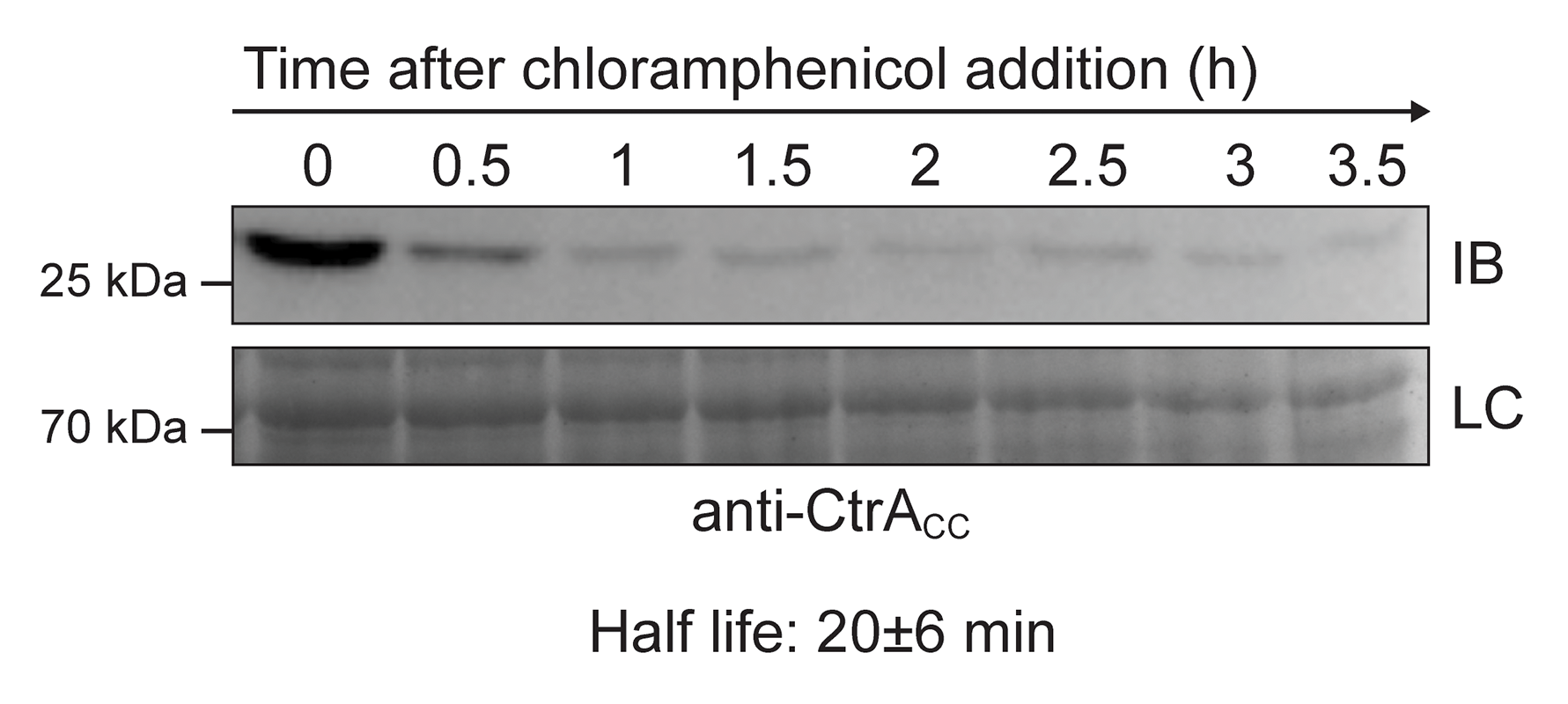

Supplement: S10 Fig — (A) Immunoblot showing decrease in the level of CtrACC in C. crescentus after the inhibition of translation. Chloramphenicol was added to an exponentially growing culture of the C. crescentus wild-type (CB15N) strain. Samples were taken at the indicated time points and probed with anti-CtrACC antibodies. Shown are a representative immunoblot (IB) and a section of the membrane stained with Amido black as a loading control (LC). To calculate the half-life of CtrACC in C. crescentus, the signals were quantified and fitted to a single-exponential function. Data represent the mean of the values obtained from two independent immunoblots (± SD). (TIF) [file pgen.1008724.s010.tif]
