# Supplementary material for: Integrative and quantitative view of the CtrA regulatory network in a stalked budding bacterium
Source: PLoS Genet. 2020 Apr 23;16(4):e1008724. doi: 10.1371/journal.pgen.1008724 (PMC7200025; doi:10.1371/journal.pgen.1008724)
Supplement: S1 Table — (PDF) [file pgen.1008724.s011.pdf]

Table S1. *E. coli* strains used in this study.

Strain	Genotype	Source
BTH101	F^- *cya-99 araD139 galK16 rpsL1 hsdR2 mcrA1 mcrB1*, Str^R	EuroMedex
Rosetta™2 (DE3)pLysS	F^- *ompT hsdSB* (rB^- mB^-) *gal dcm* (DE3) pLysSpRARE2, Cam^R	Novagen
TOP10	F^- *mcr*A Δ(*mrr-hsdRMS-mcrBC*) Φ80*lacZ*ΔM15 Δ*lac*X74 *recA*1 *ara*D139 Δ(*ara leu*) 7697 *galU galK rpsL* (Str^R) *endA*1 *nupG*	Invitrogen
WM3064	*thrB1004 pro thi rpsL hsdS lacZ*ΔM15 RP4-1360 Δ(*araBAD*)*567* Δ*dapA1341*::[*erm pir*(wt)]	W. Metcalf (unpublished data)
XL1-Blue	*recA1 endA1 gyrA96 thi-1 hsdR17 supE44 relA1 lacF' proAG laqZ M14Tn10*, Tet^R	Agilent Technologies
