# Supplementary material for: Integrative and quantitative view of the CtrA regulatory network in a stalked budding bacterium
Source: PLoS Genet. 2020 Apr 23;16(4):e1008724. doi: 10.1371/journal.pgen.1008724 (PMC7200025; doi:10.1371/journal.pgen.1008724)
Supplement: S2 Table — (PDF) [file pgen.1008724.s012.pdf]

Table S2. *H. neptunium* strains used in this study.

Strain	Genotype/description	Construction	Source
HW2	LE670 Δ*fliL*	In-frame deletion of *fliL* in LE670 using pHW6	This study
LE670	Wild type (ATCC 15444)		Leifson, 1964
OL2	LE670 P_{cckA}::P_{cckA}-*cckA-venus*	Integration of pOL2 in LE670	This study
OL20	LE670 Δ*pleC*	In-frame deletion of *pleC* in LE670 using pOL25	This study
OL21	LE670 Δ*pleD*	In-frame deletion of *pleD* in LE670 using pOL26	This study
OL23	LE670 P_{Cu}::P_{Cu}-*pleC* Δ*pleC*	Integration of pOL43 in OL20	This study
OL28	LE670 Δ*cpdR*	In-frame deletion of *cpdR* in LE670 using pOL78	This study
OL32	LE670 P_{divJ}::P_{divJ}-*divJ-venus* P_{Cu}::P_{Cu}-*mCherry-spmX*	Integration of pOL1 and pOL56 in LE670	This study
OL33	LE670 P_{pleC}::P_{pleC}-*pleC-eyfp* P_{Cu}::P_{Cu}-*mCherry-podJ*	Integration of pSW57 and pOL55 in LE670	This study
OL34	LE670 Δ*spmX*	In-frame deletion of *spmX* in LE670 using pOL58	This study
OL35	LE670 Δ*podJ*	In-frame deletion of *podJ* in LE670 using pOL59	This study
OL36	LE670 P_{divJ}::P_{divJ}-*divJ-venus* Δ*spmX*	Integration of pOL1 in LE670 and subsequent in-frame deletion of *spmX* using pOL58	This study
OL44	LE670 Δ*rcdA*	In-frame deletion of *rcdA* in LE670 using pOL85	This study
OL94	LE670 Δ*divJ*	In-frame deletion of *divJ* in LE670 using pOL24	This study
OL95	LE670 Δ*divK*	In-frame deletion of *divK* in LE670 using pOL57	This study
OL123	LE670 P_{Cu}::P_{Cu}-*divJ* Δ*divJ*	Integration of pOL167 in OL94	This study
OL146	LE670 P_{divJ}::P_{divJ}-*divJ-venus*	Integration of pOL1 in LE670	This study
OL151	LE670 P_{pleC}::P_{pleC}-*pleC-eYFP*	Integration of pSW57 in LE670	This study
OL152	LE670 P_{Cu}::P_{Cu}-*chpT* Δ*chpT*	Integration of pOL198 in LE670 and subsequent in-frame deletion of *chpT* using pOL79	This study
OL161	LE670 P_{Cu}::P_{Cu}-*cckA* Δ*cckA*	Integration of pOL203 in LE670 and subsequent in-frame deletion of *cckA* using pOL64	This study
OL166	LE670 P_{pleC}::P_{pleC}-*pleC-eyfp* Δ*podJ*	Integration of pSW57 in OL35	This study
OL177	LE670 P_{Cu}::P_{Cu}-*divL* Δ*divL*	Integration of pOL202 in LE670 and subsequent in-frame deletion of *divL* using pOL66	This study
