# Supplementary material for: Integrative and quantitative view of the CtrA regulatory network in a stalked budding bacterium
Source: PLoS Genet. 2020 Apr 23;16(4):e1008724. doi: 10.1371/journal.pgen.1008724 (PMC7200025; doi:10.1371/journal.pgen.1008724)
Supplement: S3 Table — (PDF) [file pgen.1008724.s013.pdf]

Table S3. ***C. crescentus*** **strains used in this study.**

Strain	Genotype/description	Construction	Source
CB15N	Wild type (NA1000)		Evinger & Agabian, 1977
CckATS1	CB15N $cckA_{CC}^{ts}$		Jacobs et al., 1999
KR635	CB15N $divL_{CC}^{ts}$		Reisinger et al., 2007
LS2195	CB15N $ctrA_{CC}^{ts}$		Quon et al., 1996
LS3570	CB15N $divK_{CC}^{cs}$		Hung & Shapiro, 2002
MvT81	CB15N $divL_{CC}^{ts}$ P_{xyl}::P_{xyl}-$divL_{HN}$	Integration of pMvT111 in KR635	This study
OL128	CB15N $ctrA_{CC}^{ts}$ P_{xyl}::P_{xyl}-$ctrA_{HN}$	Integration of pOL175 in LS2195	This study
OL130	CB15N P_{xyl}::P_{xyl}-$ctrA_{HN}$	Integration of pOL175 in CB15N	This study
OL133	CB15N $\Delta divJ_{CC}$::*spec* P_{xyl}::P_{xyl}-$divJ_{HN}$	Integration of pOL180 in YB3202	This study
OL135	CB15N $\Delta pleC_{CC}$ P_{xyl}::P_{xyl}-$pleC_{HN}$	Integration of pOL180 in UJ506	This study
OL137	CB15N $divK_{CC}^{cs}$ P_{xyl}::P_{xyl}-$divK_{HN}$	Integration of pOL182 in LS3570	This study
OL179	CB15N $cckA_{CC}^{ts}$ P_{xyl}::P_{xyl}-$cckA_{HN}$	Integration of pTS11 in CckATS1	This study
UJ506	CB15N $\Delta pleC_{CC}$		Aldridge et al., 2003
YB3202	CB15N $\Delta divJ_{CC}$::*spec*		Pierce et al., 2006
