# Supplementary material for: Integrative and quantitative view of the CtrA regulatory network in a stalked budding bacterium
Source: PLoS Genet. 2020 Apr 23;16(4):e1008724. doi: 10.1371/journal.pgen.1008724 (PMC7200025; doi:10.1371/journal.pgen.1008724)
Supplement: S4 Table — (PDF) [file pgen.1008724.s014.pdf]

Table S4. General plasmids used in this study.

Plasmid	Description	Source
pBBRT	pGEM-T derivative bearing *fabL* from *Bacillus subtilis*, Tcs^R, Kan^R (Promega)	Kagle & Hay, 2002
pCCYHC-2	Integrative plasmid to fuse the 3' end of a target gene to *mCherry* under the control of P_{Cu}, Kan^R	Jung et al., 2015
pCCYHC-3	Integrative plasmid to fuse the 3' end of a target gene to *mCherry* under the control of P_{Cu}, Rif^R	Jung et al., 2015
pCCYHN-3	Integrative plasmid to fuse the 5' end of a target gene to *mCherry* under the control of P_{Cu}, Rif^R	Jung et al., 2015
pCVENC-2	Integrative plasmid to fuse the 3' end of a target gene to *venus* under the control of P_{Cu}, Kan^R	Jung et al., 2015
pCVENC-8	Integrative plasmid to the fuse 3' end of a target gene to *venus* under the control of P_{Cu}, $Triclosan^R$	Leicht et al., unpublished
pET28a(+)	Plasmid for the overproduction of N-terminally His_6-tagged proteins under the control of P_{T7}, Kan^R	Novagen
pKNT25	Plasmid for bacterial two-hybrid analysis carrying the adenylate cyclase T25-subunit gene, Kan^R (Euromedex)	Battesti & Bouveret, 2012
pKNT25-zip	Control plasmid for bacterial two-hybrid analysis carrying the gene for an adenylate cyclase T25-zip fusion, Kan^R (Euromedex)	Battesti & Bouveret, 2012
pKT25	Plasmid for bacterial two-hybrid analysis carrying the adenylate cyclase T25-subunit gene, Kan^R (Euromedex)	Battesti & Bouveret, 2012
pNPTS138	*sacB*-containing suicide vector used for double-homologous recombination, Kan^R	M.R. Alley, unpublished
pTB146	Plasmid for the overproduction of N-terminally His_6-SUMO-tagged proteins under the control of P_{T7}, Amp^R	Bendezú et al., 2009
pVENC-2	Integrative plasmid to fuse the 3' end of a target gene to *venus*, Kan^R	Thanbichler et al., 2007
pUT18	Plasmid for bacterial two-hybrid analysis carrying the adenylate cyclase T18-subunit gene, Amp^R (Euromedex)	Battesti & Bouveret, 2012
pUT18-C	Plasmid for bacterial two-hybrid analysis carrying the adenylate cyclase T18-subunit gene, Amp^R (Euromedex)	Battesti & Bouveret, 2012
pUT18-C-zip	Control plasmid for bacterial two-hybrid analysis carrying carrying the gene for an adenylate cyclase T25-zip fusion, Amp^R (Euromedex)	Battesti & Bouveret, 2012
pXVENC-2	Integrative plasmid to fuse the 3' end of a target gene to *venus* under the control of P_{xyl}, Kan^R	Thanbichler et al., 2007
pXVENC-4	Integrative plasmid to fuse the 3' end of a target gene to *venus* under the control of P_{xyl}, $Gent^R$	Thanbichler et al., 2007
pYFPC-2	Integrative plasmid to fuse the 3' end of a target gene to *eyfp*, Kan^R	Thanbichler et al., 2007
