# Supplementary material for: Integrative and quantitative view of the CtrA regulatory network in a stalked budding bacterium
Source: PLoS Genet. 2020 Apr 23;16(4):e1008724. doi: 10.1371/journal.pgen.1008724 (PMC7200025; doi:10.1371/journal.pgen.1008724)
Supplement: S5 Table — (PDF) [file pgen.1008724.s015.pdf]

Table S5. Plasmids generated in this study.

Plasmid	Description	Construction
pCVENC-8	pCVENC-2 derivative a carrying triclosan-resistance cassette	a) PCR amplification of *fabL* from pBBRT with primers oOL694 and oOL695<br>b) Annealing of oligonucleotides oOL692 and oOL693<br>c) Fusion of the fragment and the dimerized oligonucleotides with pCVENC-2 via Gibson assembly
pHW6	pNPTS138 derivative for generating an in-frame deletion of *fliL*$_{HN}$	a) PCR amplification of a fragment containing 600 bp of the *fliL*$_{HN}$ upstream region and the first 36 bp of *fliL*$_{HN}$ with primers oHW1 and oHW2<br>b) PCR amplification of a fragment containing 600 bp of the *fliL*$_{HN}$ downstream region and the last 36 bp of *fliL*$_{HN}$ with primers oHW3 and oHW4<br>c) Overlap-extension PCR to fuse both fragments using the primers oHW1 and oHW4<br>d) Digestion of the product with HindIII and NheI<br>e) Ligation of the two fragments into HindIII/NheI-treated pNPTS138
pJR74	pTB146 bearing HNE_0264 (encoding flagellin)	a) PCR amplification of HNE_0264 with primers oJR52 and oOL53<br>b) Digestion of the product with SacI and BamHI<br>c) Ligation of the fragment into SacI/BamHI-treated pTB146
pJR75	pET28a(+) bearing *cckA*$_{HN\ AA734\text{-}867}$	a) PCR amplification of with primers oJR62 *cckA*$_{HN\ AA734\text{-}867}$ and oJR63<br>b) Digestion of the product with BamHI and NdeI<br>c) Ligation of the fragment into BamHI/NdeI-treated pET28a(+)
pMSB2	pET28a(+) bearing *cckA*$_{AA61\text{-}571}$	a) PCR amplification of *cckA*$_{CC\ AA61\text{-}571}$ with primers oMSB1 and oMSB4<br>b) Fusion of the fragment with BamHI/NdeI-treated pET28a(+) via Gibson assembly
pMSB3	pET28a(+) bearing *cckA*$_{AA61\text{-}691}$	a) PCR amplification of *cckA*$_{CC\ AA61\text{-}691}$ with primers oMSB1 and oMSB2<br>b) Fusion of the fragment with BamHI/NdeI-treated pET28a(+) via Gibson assembly
pMvT111	pXVENC-4 bearing *divL*$_{HN}$	a) PCR amplification of *divL*$_{HN}$ including stop-codon with primers oMvT622 and oMvT623<br>b) Fusion of the fragment with MluI/NdeI-treated pXVENC-4
pOL1	pVENC-2 bearing *divJ*$_{HN}$	a) PCR amplification of the last 607 bp of *divJ*$_{HN}$ excluding stop-codon with primers oJK179 and oJK180<br>b) Digestion of the product with NdeI and EcoRI<br>c) Ligation of the fragment into NdeI/EcoRI-treated pVENC-2
pOL2	pVENC-2 bearing *cckA*$_{HN}$	a) PCR amplification of the last 613 bp of *cckA*$_{HN}$ excluding stop-codon with primers oJK177 and oJK178<br>b) Digestion of the product with NdeI and EcoRI<br>c) Ligation of the fragment into NdeI/EcoRI-treated pVENC-2

pOL24	pNPTS138 derivative for generating an in-frame deletion of *divJ*$_{HN}$	a) PCR amplification of a fragment containing 601 bp of the *divJ*$_{HN}$ upstream region and the first 36 bp of *divJ*$_{HN}$ with primers oOL37 and oOL38<br>b) Digestion of the product with SpeI and EcoRI<br>c) PCR amplification of a fragment containing 600 bp of the *divJ*$_{HN}$ downstream region and the last 36 bp of *divJ*$_{HN}$ with primers oOL39 and oOL40<br>d) Digestion of the product with EcoRI and PstI<br>e) Ligation of the two fragments into SpeI/EcoRI-treated pNPTS138
pOL25	pNPTS138 derivative for generating an in-frame deletion of *pleC*$_{HN}$	a) PCR amplification of a fragment containing 600 bp of the *pleC*$_{HN}$ upstream region and the first 36 bp of *pleC*$_{HN}$ with primers oOL41 and oOL42<br>b) PCR amplification of a fragment containing 601 bp of the *pleC*$_{HN}$ downstream region and the last 36 bp of *pleC*$_{HN}$ with primers oOL43 and oOL44<br>c) Overlap-extension PCR to fuse both fragments using the primers oOL41 and oOL44<br>d) Digestion of the product with SpeI and EcoRI<br>e) Ligation of the two fragments into SpeI/EcoRI-treated pNPTS138
pOL26	pNPTS138 derivative for generating an in-frame deletion of *pleD*$_{HN}$	a) PCR amplification of a fragment containing 600 bp of the *pleD*$_{HN}$ upstream region and the first 36 bp of *pleD*$_{HN}$ with primers oOL45 and oOL46<br>b) PCR amplification of a fragment containing 601 bp of the *pleD*$_{HN}$ downstream region and the last 36 bp of *pleD*$_{HN}$ with primers oOL47 and oOL48<br>c) Overlap-extension PCR to fuse both fragments using the primers oOL45 and oOL48<br>d) Digestion of the product with SpeI and HindIII<br>e) Ligation of the two fragments into SpeI/HindIII-treated pNPTS138
pOL33	pKNT25 bearing *divJ*$_{HN}$	a) PCR amplification of *divJ*$_{HN}$ with primers oOL88 and oOL89<br>b) Digestion of the product with KpnI and EcoRI<br>c) Ligation of the fragment into KpnI/EcoRI-treated pKNT25
pOL34	pUT18 bearing *divJ*$_{HN}$	a) PCR amplification of *divJ*$_{HN}$ with primers oOL88 and oOL89<br>b) Digestion of the product with KpnI and EcoRI<br>c) Ligation of the fragment into KpnI/EcoRI-treated pUT18
pOL35	pKNT25 bearing *pleC*$_{HN}$	a) PCR amplification of *pleC*$_{HN}$ with primers oOL92 and oOL93<br>b) Digestion of the product with KpnI and EcoRI<br>c) Ligation of the fragment into KpnI/EcoRI-treated pKNT25
pOL36	pUT18 bearing *pleC*$_{HN}$	a) PCR amplification of *pleC*$_{HN}$ with primers oOL92 and oOL93<br>b) Digestion of the product with KpnI and EcoRI<br>c) Ligation of the fragment into KpnI/EcoRI-treated pUT18

pOL37	pUT18-C bearing *divK*$_{HN}$	a) PCR amplification of *divK*$_{HN}$ with primers oOL86 and oOL87<br>b) Digestion of the product with KpnI and EcoRI<br>c) Ligation of the fragment into KpnI/EcoRI-treated pUT18-C
pOL38	pKT25 bearing *divK*$_{HN}$	a) PCR amplification of *divK*$_{HN}$ with primers oOL86 and oOL87<br>b) Digestion of the product with KpnI and EcoRI<br>c) Ligation of the fragment into KpnI/EcoRI-treated pKT25
pOL43	pCCHYC-3 bearing *pleC*$_{HN}$	a) PCR amplification of *pleC*$_{HN}$ including stop-codon with primers oOL99 and oOL101<br>b) Digestion of the product with NdeI and KpnI<br>c) Ligation of the fragment into NdeI/KpnI-treated pCCHYC-3
pOL47	pKNT25 bearing *divL*$_{HN}$	a) PCR amplification of *divL*$_{HN}$ with primers oOL108 and oOL109<br>b) Digestion of the product with KpnI and EcoRI<br>c) Ligation of the fragment into KpnI/EcoRI-treated pKNT25
pOL48	pUT18 bearing *divL*$_{HN}$	a) PCR amplification of *divL*$_{HN}$ with primers oOL108 and oOL109<br>b) Digestion of the product with KpnI and EcoRI<br>c) Ligation of the fragment into KpnI/EcoRI-treated pUT18
pOL55	pCCHYN-3 bearing *podJX*$_{HN}$	a) PCR amplification of *podJ*$_{HN}$ including stop-codon with primers oOL31 and oOL32<br>b) Digestion of the product with KpnI and NheI<br>c) Ligation of the fragment into KpnI/NheI-treated pCCHYC-3
pOL56	pCCHYN-3 bearing *spmX*$_{HN}$	a) PCR amplification of *spmX*$_{HN}$ including stop-codon with primers oOL33 and oOL34<br>b) Digestion of the product with KpnI and NheI<br>c) Ligation of the fragment into KpnI/NheI-treated pCCHYC-3
pOL57	pNPTS138 derivative for generating an in-frame deletion of *divK*$_{HN}$	a) PCR amplification of a fragment containing 600 bp of the *divK*$_{HN}$ upstream region and the first 36 bp of *divK*$_{HN}$ with primers oOL115 and oOL116<br>b) PCR amplification of a fragment containing 605 bp of the *divK*$_{HN}$ downstream region and the last 36 bp of *divK*$_{HN}$ with primers oOL117 and oOL118<br>c) Overlap-extension PCR to fuse both fragments using the primers oOL115 and oOL118<br>d) Digestion of the product with SpeI and EcoRI<br>e) Ligation of the two fragments into SpeI/EcoRI-treated pNPTS138
pOL58	pNPTS138 derivative for generating an in-frame deletion of *spmX*$_{HN}$	a) PCR amplification of a fragment containing 600 bp of the *spmX*$_{HN}$ upstream region and the first 36 bp of *spmX*$_{HN}$ with primers oOL125 and oOL126<br>b) PCR amplification of a fragment containing 600 bp of the *spmX*$_{HN}$ downstream region and the last 36 bp of *spmX*$_{HN}$ with primers oOL127 and oOL128<br>c) Overlap-extension PCR to fuse both fragments using the primers oOL125 and oOL128<br>d) Digestion of the product with SpeI and EcoRI

		Ligation of the two fragments into SpeI/EcoRI-treated pNPTS138
pOL59	pNPTS138 derivative for generating an in-frame deletion of *podJ*$_{HN}$	a) PCR amplification of a fragment containing 600 bp of the *podJ*$_{HN}$ upstream region and the first 36 bp of *podJ*$_{HN}$ with primers oOL131 and oOL132<br>b) PCR amplification of a fragment containing 600 bp of the *podJ*$_{HN}$ downstream region and the last 36 bp of *podJ*$_{HN}$ with primers oOL133 and oOL134<br>c) Overlap-extension PCR to fuse both fragments using the primers oOL131 and oOL134<br>d) Digestion of the product with SpeI and EcoRI<br>e) Ligation of the two fragments into SpeI/EcoRI-treated pNPTS138
pOL64	pNPTS138 derivative for generating an in-frame deletion of *cckA*$_{HN}$	a) PCR amplification of a fragment containing 602 bp of the *cckA*$_{HN}$ upstream region and the first 36 bp of *cckA*$_{HN}$ with primers oOL154 and oOL155<br>b) PCR amplification of a fragment containing 600 bp of the *cckA*$_{HN}$ downstream region and the last 36 bp of *cckA*$_{HN}$ with primers oOL156 and oOL157<br>c) Overlap-extension PCR to fuse both fragments using the primers oOL154 and oOL157<br>d) Digestion of the product with SpeI and EcoRI<br>e) Ligation of the two fragments into SpeI/EcoRI-treated pNPTS138
pOL66	pNPTS138 derivative for generating an in-frame deletion of *divL*$_{HN}$	a) PCR amplification of a fragment containing 603 bp of the *divL*$_{HN}$ upstream region and the first 36 bp of *divL*$_{HN}$ with primers oOL162 and oOL163<br>b) PCR amplification of a fragment containing 600 bp of the *divL*$_{HN}$ downstream region and the last 36 bp of *divL*$_{HN}$ with primers oOL164 and oOL165<br>c) Overlap-extension PCR to fuse both fragments using the primers oOL162 and oOL165<br>d) Digestion of the product with SpeI and EcoRI<br>e) Ligation of the two fragments into SpeI/EcoRI-treated pNPTS138
pOL78	pNPTS138 derivative for generating an in-frame deletion of *cpdR*$_{HN}$	a) PCR amplification of a fragment containing 602 bp of the *cpdR*$_{HN}$ upstream region and the first 36 bp of *cpdR*$_{HN}$ with primers oOL174 and oOL175<br>b) PCR amplification of a fragment containing 601 bp of the *cpdR*$_{HN}$ downstream region and the last 36 bp of *cpdR*$_{HN}$ with primers oOL176 and oOL177<br>c) Overlap-extension PCR to fuse both fragments using the primers oOL174 and oOL177<br>d) Digestion of the product with SpeI and EcoRI<br>e) Ligation of the two fragments into SpeI/EcoRI-treated pNPTS138

pOL79	pNPTS138 derivative for generating an in-frame deletion of *chpT*$_{HN}$	a) PCR amplification of a fragment containing 600 bp of the *chpT*$_{HN}$ upstream region and the first 36 bp of *chpT*$_{HN}$ with primers oOL282 and oOL283<br>b) PCR amplification of a fragment containing 600 bp of the *chpT*$_{HN}$ downstream region and the last 36 bp of *chpT*$_{HN}$ with primers oOL284 and oOL285<br>c) Overlap-extension PCR to fuse both fragments using the primers oOL282 and oOL285<br>d) Digestion of the product with SpeI and EcoRI<br>e) Ligation of the two fragments into SpeI/EcoRI-treated pNPTS138
pOL85	pNPTS138 derivative for generating an in-frame deletion of *rcdA*$_{HN}$	a) PCR amplification of a fragment containing 600 bp of the *rcdA*$_{HN}$ upstream region and the first 36 bp of *rcdA*$_{HN}$ with primers oOL288 and oOL289<br>b) PCR amplification of a fragment containing 651 bp of the *rcdA*$_{HN}$ downstream region and the last 36 bp of *rcdA*$_{HN}$ with primers oOL290 and oOL300<br>c) Overlap-extension PCR to fuse both fragments using the primers oOL288 and oOL300<br>d) Digestion of the product with SpeI and EcoRI<br>e) Ligation of the two fragments into SpeI/EcoRI-treated pNPTS138
pOL134	pET28a(+) bearing *divK*$_{HN}$	a) PCR amplification of *divK*$_{HN}$ with primers oOL581 and oOL582<br>b) Digestion of the product with HindIII and NdeI<br>c) Ligation of the fragment into HindIII/NdeI-treated pET28a(+)
pOL135	pET28a(+) bearing *divJ*$_{HN, AA280-539}$	a) PCR amplification of *divJ*$_{HN, AA280-539}$ with primers oOL585 and oOL586<br>b) Digestion of the product with HindIII and NdeI<br>c) Ligation of the fragment into HindIII/NdeI-treated pET28a(+)
pOL145	pET28a(+) bearing *ctrA*$_{HN}$	a) PCR amplification of *ctrA*$_{HN}$ with primers oOL614 and oOL261<br>b) Digestion of the product with EcoRI and NdeI<br>c) Ligation of the fragment into EcoRI/NdeI-treated pET28a(+)
pOL167	pCCHYC-2 bearing *divJ*$_{HN}$	a) PCR amplification of *divJ*$_{HN}$ including stop-codon with primers oOL637 and oOL638<br>b) Digestion of the product with EcoRI and NdeI<br>c) Ligation of the fragment into EcoRI/NdeI-treated pCCHYC-2
pOL175	pXVENC-2 bearing *ctrA*$_{HN}$	a) PCR amplification of *ctrA*$_{HN}$ including stop-codon with primers oOL665 and oOL666<br>b) Digestion of the product with EcoRI and NdeI<br>c) Ligation of the fragment into EcoRI/NdeI-treated pXVENC-2
pOL180	pXVENC-2 bearing *divJ*$_{HN}$	a) PCR amplification of *divJ*$_{HN}$ including stop-codon with primers oOL674 and oOL638<br>b) Digestion of the product with NdeI and EcoRI<br>c) Ligation of the fragment into NdeI/EcoRI-treated pXVENC-2

pOL181	pXVENC-2 bearing *pleC*$_{HN}$	a) PCR amplification of *pleC*$_{HN}$ including stop-codon with primers oOL676 and oOL690<br>b) Digestion of the product with NdeI and EcoRI<br>c) Ligation of the fragment into NdeI/EcoRI-treated pXVENC-2
pOL182	pXVENC-2 bearing *divK*$_{HN}$	a) PCR amplification of *divK*$_{HN}$ including stop-codon with primers oOL750 and oOL691<br>b) Digestion of the product with EcoRI and NdeI<br>c) Ligation of the fragment into EcoRI/NdeI-treated pXVENC-2
pOL198	pCVENC-8 bearing *chpT*$_{HN}$	a) PCR amplification of *chpT*$_{HN}$ with primers oOL721 and oOL722<br>b) Digestion of the product with EcoRI and NdeI<br>c) Ligation of the fragment into EcoRI/NdeI-treated pCVENC-8
pOL202	pCVENC-8 bearing *divL*$_{HN}$	a) PCR amplification of *divL*$_{HN}$ with primers oOL729 and oOL730<br>b) Fusion of the fragment with EcoRV-treated pCVENC-8 via Gibson Assembly
pOL203	pCVENC-8 bearing *cckA*$_{HN}$	a) PCR amplification of *cckA*$_{HN}$ with primers oOL727 and oOL728<br>b) Fusion of the fragment with EcoRV-treated pCVENC-8 via Gibson Assembly
pOL207	pET28a(+) bearing *chpT*$_{HN}$	a) PCR amplification of *chpT*$_{HN}$ with primers oOL740 and oOL741<br>b) Digestion of the product with HindIII and NdeI<br>c) Ligation of the fragment into HindIII/NdeI-treated pET28a(+)
pOL208	pET28a(+) bearing *pleC*$_{HN,\ AA565\text{-}821}$	a) PCR amplification of *pleC*$_{HN,\ AA565\text{-}821}$ with primers oOL742 and oOL743<br>b) Digestion of the product with NdeI and HindIII<br>c) Ligation of the fragment into NdeI/HindIII-treated pET28a(+)
pOL209	pTB146 bearing *ctrA*$_{HN}$	a) PCR amplification of *ctrA*$_{HN}$ with primers oOL744 and oOL745<br>b) Digestion of the product with EcoRV<br>c) Ligation of the fragment into EcoRV-treated pTB146
pSW57	pYFPC-2 bearing *pleC*$_{HN}$	a) PCR amplification of *pleC*$_{HN}$ with primers oSW114 and oSW115<br>b) Digestion of the product with NdeI and EcoRI<br>c) Ligation of the fragment into NdeI/EcoRI-treated pYFPC-2
pTS11	pXVENC-2 bearing *cckA*$_{HN}$	a) PCR amplification of *cckA*$_{HN}$ including stop-codon with primers oTS23 and oTS24<br>b) Digestion of the product with NdeI and KpnI<br>c) Ligation of the fragment into NdeI/KpnI-treated pXVENC-2
