# Supplementary material for: Integrative and quantitative view of the CtrA regulatory network in a stalked budding bacterium
Source: PLoS Genet. 2020 Apr 23;16(4):e1008724. doi: 10.1371/journal.pgen.1008724 (PMC7200025; doi:10.1371/journal.pgen.1008724)
Supplement: S6 Table — (PDF) [file pgen.1008724.s016.pdf]

Table S6. **Oligonucleotides in this study.**

Oligo-nucleotide	Name	Sequence
oHW1	HNE_0264 P1	ATAAAGCTTCCGAAACGCATCCGCTGG
oHW2	HNE_0264 P2	TTAGCGGAAGAGCGACAGAGTCAGGATGCTAGACAT
oHW3	HNE_0264 P3	ATGTCTAGCATCCTGACTCTGTCGCTCTTCCGCTAA
oHW4	HNE_0264 P4	ATAGCTAGCCCTCTTTGGGATGCATCG
oJK177	HNE_0507s for NdeI	ATATCATATGCCAGCCTTTCTTCACCACGAAAG
oJK178	HNE_0507 rev EcoRI	ATATGAATTCGGCGCGGCTTCCTTGCC
oJK179	HNE_0746s for NdeI	ATATCATATGGGGCCCGATGCCGGAAGTC
oJK180	HNE_0746 rev EcoRI	ATATGAATTCAATTCGGCGGCTTCGTCGAGC
oJR52	HNE_0264-SacI-for	TATAGAGCTCATGTCTAGCATCCTGACTAACAACAG
oJR53	HNE_0264-BamHI-rev	TATAGGATCCTTAGCGGAAGAGCGACAGCAGAGC
oJR62	HNE_0507 short-for	TTTTCATATGGTCCGCCCGATGGACATGTCCGGGC
oJR63	HNE_0507 short-rev	TTGGATCCTCACGCGGCTTCCTTGCCGCCGATG
oMSB1	CCNA-01132-KD_F	CTGGTGCCGCGCGGCAGCCAACGCGGCTCAGCGCTTTCCGG
oMSB2	CCNA-01132-KD_R	GACGGAGCTCGAATTCGCTAGGCGCCCGACAGGTCGCGAGCGG
oMSB4	CCNA-01132-RR_R	GTCGACGGAGCTCGAATTCGCTACGCCGCCTGCAGCTGCTG
oMvT622	HNE_0399_p1	GGGGAGACGACCATATGTATGACCGCTGAACAGATGCCC
oMvT623	HNE_0399_p2	GGTGGCCGACCGGTGACGCGTTAACGTTCGAATTCTCAGTGCGCCG
oOL31	HNE_0666_F1	TAGGTACCATGAGCCAGACGGGGCCCTGGAGC
oOL32	HNE_0666_R1	ATATGCTAGCTCAGCCCCGGCGGTTTGACGCGC
oOL33	HNE_1271_F1	TAGGTACCATGGGTGGTCCGCTTCGCACATCTG
oOL34	HNE_1271_R1	ATATGCTAGCTCACTCGTCCTGCGTCATCGCCCG
oOL37	HNE_0746_Del_P1	ATATACTAGTCCCTGTTCCTTGACGATGGCCGCG
oOL38	HNE_0746_Del_P2	CAGATCCGCAAACTGTAATCGCTTCTGCGACATGCGG
oOL39	HNE_0746_Del_P3	TTACAGTTTGCGGATCTGTTGCCGCTCGACGAAG
oOL40	HNE_0746_Del_P4	ATGAATTCTTCATGGAGGGCACATAGGCGGCCG
oOL41	HNE_2910_Del_P1	ATATACTAGTCCCGGCTATTTATCGTCCGGCACGG
oOL42	HNE_2910_Del_P2	CCTATTTCCGCGGTCCGCTTCGACATCCAGCGG
oOL43	HNE_2910_Del_P3	GCGGACCGCGGAAATAGGCAGCCTTACCTTTTCCCC
oOL44	HNE_2910_Del_P4	ATGAATTCGCTCAGCCTTCATCCTGCTGCTGGG
oOL45	HNE_2284_Del_P1	ATATACTAGTGCAAAGAGCGTCGTGGCGGTGCCATAG
oOL46	HNE_2284_Del_P2	GACATCGAGGGCGGCCGGAACCGCGTCGAGAGC
oOL47	HNE_2284_Del_P3	CCGGCCGCCCTCGATGTCGTCCACGACGAGGATGCG
oOL48	HNE_2284_Del_P4	ATATAAGCTTGGCTGCCACAGGCGGGGCAGGCGG
oOL86	HNE_2285_Bacth_For	TAGGTACCGATGATGGTCAAGAAGCGATCCGGAAA
oOL87	HNE_2285_Bacth_Rev	ATGAATTCTCATGCCGCCGGTTCCTTGGGCATCA
oOL88	HNE_0746_Bacth_For	TAGGTACCATGGACCCCCGCATGTCGCAGAA
oOL89	HNE_0746_Bacth_Rev	ATGAATTCTTTTCGGCGGCTTCGTCGAGCG
oOL92	HNE_2910_Bacth_For	TAGGTACCATGATTCGCGGGGAAAAGGTAAGGCT

oOL93	HNE_2910_Bacth_Rev	ATGAATTCCTGGCAACATCCCGCTGGATGTCGA
oOL99	HNE_2910_Comp_For	ATTAATTCATATGATTCGCGGGGAAAAGGTAAGGCT
oOL100	HNE_2910_Comp_Rev_Stop	TAGGTACCGGCAACATCCCGCTGGATGTCGAA
oOL108	HNE_0399_Bacth_For	ATGGTACCATGACCGCTGAACAGATGCCCATT
oOL109	HNE_0399_Bacth_Rev	ATGAATTCGGGTGCGCCGTGGGCTGGGGCT
oOL115	HNE_2285_Del_P1	ATATACTAGTGGATCAGCACGTCGACACGCTCGGT
oOL116	HNE_2285_Del_P2	GTGCTTGTTGTTGAAGCGCTGATGCCCAAGGAA
oOL117	HNE_2285_Del_P3	CGCTTCAACAACAAGCACCTTTCCGGATCGCTTCT
oOL118	HNE_2285_Del_P4	ATGAATTCGCTGGCAGCCGGCGGGGCGCCGT
oOL125	HNE_1271_Del_P1	ATATACTAGTCCGTGGCTGGGCGTGAACCT
oOL126	HNE_1271_Del_P2	ATACGTGGCGAGTCCCTTTGCAGATGTGCGAA
oOL127	HNE_1271_Del_P3	AAGGGACTCGCCACGTATTACCTTTACCGGGC
oOL128	HNE_1271_Del_P4	ATGAATTCGCCGCCATTCGGGTCGGT
oOL131	HNE_0666_Del_P1	ATATACTAGTGTGCCGGTAGATGCGCCTGCT
oOL132	HNE_0666_Del_P2	AAGGGAATCGATCGACCGCCTCAACACCGGC
oOL133	HNE_0666_Del_P3	CGGTCGATCGATTCCCTTAACGCTCCAGGGCC
oOL134	HNE_0666_Del_P4	ATGAATTCCTTCGAGTTCGCGGGCCTGCT
oOL154	HNE_0507_Del_P1	ATAACTAGTGAAGGTGTAGCTGTGGTGAGGGAA
oOL155	HNE_0507_Del_P2	GGCCAGTTCGAGTGTCAGCTCTAGGGTGTCCGC
oOL156	HNE_0507_Del_P3	CTGACACTCGAACTGGCCGGCATCGGCG
oOL157	HNE_0507_Del_P4	ATAGAATTCCTGGCTGATCACCCAGATGGAAG
oOL162	HNE_0399_Del_P1	ATAACTAGTGCGGTCAGCTCGCCATCGC
oOL163	HNE_0399_Del_P2	CGCGCTTTGAATAGCCATCAGAATGGGCATCTGT
oOL164	HNE_0399_Del_P3	ATGGCTATTCAAAGCGCGTTCGAGCCCCA
oOL165	HNE_0399_Del_P4	ATAGAATTCCAAGCCGATTGCCTCCTCGG
oOL174	HNE_0229_Del_P1	ATAACTAGTCTCCGCCTTGTACAGGAGCC
oOL175	HNE_0229_Del_P2	TTCCACGATCGAATCATCATCTTCAGCGAGC
oOL176	HNE_0229_Del_P3	GATGATTCGATCGTGGAAGAGGTAGACAAGGTTA
oOL177	HNE_0229_Del_P4	ATAGAATTCCCGTCACAAGGCTGGAACG
oOL261	6xHis_HNE_0944_rev	ATAGAATTCTCAGGCGGCTTCTTCGATCTC
oOL282	HNE_0638_Del_P1	ATAACTAGTCTGGCGAAACCGGAACGACC
oOL283	HNE_0638_Del_P2	CATGAACACGATAAATGCAGCGAAACGCGC
oOL284	HNE_0638_Del_P3	GCATTTATCGTGTTCATGGCCACCGGCCTCAA
oOL285	HNE_0638_Del_P4	ATAGAATTCGCGCGTGACGGCGGGATTGC
oOL288	HNE_0013_Del_P1	ATAACTAGTCTTGAAACGTGACGTGCCAGGG
oOL289	HNE_0013_Del_P2	CGGATCGAACAGCGACTGCGGGGAAACCA
oOL290	HNE_0013_Del_P3	CAGTCGCTGTTCGATCCGCTGATGATCTGGC
oOL300	HNE_0013_Del_P4_New	ATAGAATTCCATGACAACGCCGACCGAAA
oOL581	6xHis_HNE_2285_For	ATACATATGTTGATGGTCAAGAAGCGATC
oOL582	6xHis_HNE_2285_Rev	ATAAAGCTTTCATGCCGCCGGTTCCTTGG
oOL585	6xHis_HNE_0746_aa283_For	ATACATATGTTTGCGCCGCCGGAGACCGT
oOL586	6xHis_HNE_0746_aa283_Rev	ATAAAGCTTCTATTCGGCGGCTTCGTCGA

oOL614	6xHis_HNE_0944_for	ATACATATGCGCGTCCTGCTAATTGAAGAT
oOL637	HNE_0746_For	ATACATATGGACCCCCGCATGTCGCAGAA
oOL638	HNE_0746_Rev	TATGAATTCCTATTCGGCGGCTTCGTCGA
oOL665	HNE_0944_For	ATACATATGCGCGTCCTGCTAATTGAAGAT
oOL666	HNE_0944_Rev	TATGAATTCTCAGGCGGCTTCTTCGATCTC
oOL674	HNE_0746_For_PCVENC_2	ATATCATATGGACCCCCGCATGTCGCAGAAGCG
oOL676	HNE_2910_For_PCVENC_2	ATATCATATGATTCGCGGGGAAAAGGTAAGGC
oOL690	HNE_2910_Rev_Stop_EcoRI	ATATGAATTCTCAGGCAACATCCCGCTGGATGTCGAA
oOL691	HNE_2285_Rev	ATATATGAATTCTCATGCCGCCGGTTCCTTGGGCAT
oOL692	Prom_0038_15bp_Overlap_GibsonDS_For_Comp	AACCAATTCTGATTAACCAAAACCTCGAGAGACAAGACTTACAGGAGAACCCCCCATGGAACAAAATAAA
oOL693	Prom_0038_15bp_Overlap_GibsonDS_Rev_Comp	TTTATTTTGTTCCATGGGGGGTTCTCCTGTAAGTCTTGTCTCTCGAGGTTTTGGTTAATCAGAATTGGTT
oOL694	FabL_For_Overlap_Prom0038_GibsonSS	ACAGGAGAACCCCCCATGGAACAAAATAAATGTGC
oOL695	FabL_Rev_Overlap_PCVENC_2_GibsonSS	AATACAAGGGGTGTTTTAAACGAGCAGTGAGCGTC
oOL696	PVENC_2_Rev_Overlap_Prom_0038_Gibson_SS	TCTCGAGGTTTTGGTTAATCAGAATTGGTTAATTG
oOL697	PVENC_2_For_Overlap_FabL_Gibson_SS	TCACTGCTCGTTTAAAACACCCCTTGTATTACTGT
oOL721	HNE_0638_For	ATATCATATGAACCTTGATCATGCGCGTTT
oOL722	HNE_0638_Rev	ATATGAATTCTCAGGCTTGCGGTTTGAGGC
oOL727	HNE_0507_For	CATTCACAGGAACTCTTCCATATGAGTATTGATGCGGACACCCTAGAG
oOL728	HNE_0507_Rev	AATTAAGGCGCCTGCAGGCATATGTCACGCGGCTTCCTTGCC
oOL729	HNE_0399_For	CATTCACAGGAACTCTTCCATATGACCGCTGAACAGATGCCC
oOL730	HNE_0399_Rev	AATTAAGGCGCCTGCAGGCATATGTCAGTGCGCCGTGGGC
oOL740	6xHis_HNE_0638_for	ATATCATATGAACCTTGATCATGCGCGTTT
oOL741	6xHis_HNE_0638_rev	ATATAAGCTTTCAGGCTTGCGGTTTGAGGC
oOL742	6xHis_HNE_2910_For	ATATCATATGCGAAAGTATTCGGAGGAAAA
oOL743	6xHis_HNE_2910_Rev	ATATAAGCTTTCAGGCAACATCCCGCTGGA
oOL744	HNE_0944_For_His_SUMO_GA	ACAGAGAACAGATTGGTGGTATGCGCGTCCTGCTAATTGA
oOL745	HNE_0944_Rev_His_SUMO_GA	CGACGGAGCTCTGCTCTTCTTCAGGCGGCTTCTTCGATCT
oOL750	HNE_2285_For	ATATCATATGATGGTCAAGAAGCGATCC
oSW114	HNE2910-for	TTAACATATGTCATGACCAACGAGCTTGCCGGACC
oSW115	HNE2910-rev	TAGAATTCGAGGCAACATCCCGCTGGATGTCGAAG
oTS23	HNE_0507_for	ATACATATGAGTATTGATGCGGACACCCT
oTS24	HNE_0507_rev	ATAGGTACCTCACGCGGCTTCCTTGCCGC
