# Supplementary material for: Integrative and quantitative view of the CtrA regulatory network in a stalked budding bacterium
Source: PLoS Genet. 2020 Apr 23;16(4):e1008724. doi: 10.1371/journal.pgen.1008724 (PMC7200025; doi:10.1371/journal.pgen.1008724)
Supplement: Attachment — Submitted filename: Response to reviewers.docx [file pgen.1008724.s032.docx]

**Response to reviewers**

**We thank the three reviewers for their constructive criticism, which has helped to significantly improve our paper. Please see below for our responses to the issues raised.**

**Reviewer #1:**

Sequencing of many bacterial genomes will undoubtly generate questions about the extend of extrapolations we can make for functions in diverse bacteria, based on homology with model species. Therefore the study reported here by the Thanbichler team is really important for the field of molecular microbiology. The topic is very interesting and the conclusions are well supported by the numerous data included in this manuscript. It is an impressive work of high quality and the manuscript is well written. I have several comments and requests for a revised version of the manuscript.

The abstract is too vague and too general, and should be written with more precision, for example reporting that divK is not essential, that CckA localization is completely different from the one in C. crescentus and that CtrA half life is much longer...

**We have changed the abstract to include more detailed information. All findings pointed out by the reviewer have now been described in more detail.**

Line 114 (Table 1) : I would include the E-value of the next (i.e. second) closest homolog of each C. crescentus protein in H. netunium genome, to really show that the identified H. neptunium is probably an ortholog. Also, are they best reciprocal matches?

**We have now included these data in Table 1.**

Line 123 (Figure 3B and 3C) : I suggest to add the statistical analysis of the data to compare the swarm area of the different strains

**Changed accordingly.**

Line 146 (Figure S3) : it is not obvious that DNA is segregated to the nascent daughter cell for the ∆cckA PCu-cckA uninduced condition. Is it also true for only one bacterium in the ∆chpT PCu-chpT uninduced strain? It would be nice to show more bacteria or to have a quantification.

**We have now included the results of the suggested quantification as part of Fig. S3.**

Lines from 155 (Figure 5A,B,C,D) : what is the proportion of bacteria with a polar fusion to DivJ, SpmX, PleC or PodJ? I think that this data should be incorporated.

**We have now included an analysis showing these data as part of Fig. 5.**

Line 166 (Figure 5E,F) : does CckA contain one or several predicted transmembrane segments like in C. crescentus?

**Like its homolog in *C. crescecentus*, CckA_HNE_ contains two predicted transmembrane domains. This information is now included in the revised manuscript (L174-175).**

Line 184: I think that reference to Figure S1A is wrong here

**We intended to refer to a figure showing that CckA_CC_ is functionally equivalent to CckA_HNE_, which is shown in Fig. S1A. We have now moved the reference to a more suitable position in the sentence to avoid confusion. See L195 of the revised manuscript.**

Line 187 : Figure S7 mentions that ChpT is dispensable if CckARR is absent. I think that this information should be removed because, in the current state of the manuscript, it does not bring a new interesting data

**We agree with the reviewer that this finding does not provide any interesting new insights on its own. The direct transfer of a phosphoryl group from the isolated CckA HK domain to CtrA is unlikely to be physiologically relevant. Nevertheless, this experiment should be included in the supplements, as it is an important control for our phosphotransfer experiments.**

Line 195 : Figure 8A,H : I would remove the WT line (not necessary)

**We do agree with the reviewer that the black lines are not 100% necessary, but we feel they aid the reader by defining a clear baseline. As Fig. 8 is already relatively complex, we would prefer to keep the black lines.**

Line 197 : the authors report genes up- or down-regulated in the absence of CckA and/or ChpT as "the global CtrA regulon". I think that this is not correct, because CckA and ChpT could control other (unknown) regulators.

**We agree with the reviewer’s words of caution and have clarified this in the manuscript (see L210-211 of the revised manuscript).**

Line 201 : 286 genes are mentionned here while it is 284 genes in Figure 8F. In Figure 8F I would indicate the numbers 381 and 284 (or 286) outside the Venn ensemble, while keeping "94" in the intersection between these ensembles.

**Thank you for pointing out this mistake! We have now corrected this numbering throughout the manuscript. We have changed the Venn diagram according to the comment.**

Line 214 : in Figure 8B,E,G would it possible to place the COGs in the same order? indeed, several COGs are shown in black, and it is thus difficult to know which fraction corresponds to which COG.

**We agree with the reviewer that some COGs had a very similar hue in the original manuscript. We have now adjusted the colors to increase the clarity of the figure. However, we would like to keep the order of the overarching categories (and the COGs in each category) in the original order, because it indicates the extent to which the different functions are represented in each dataset. As requested by reviewer 3, the pie charts are now placed in a new supplementary figure (Fig S9).**

Line 221 : reference to Figure 8G should be Figure 8H?

**Thank you for pointing out this mistake! We have changed the reference to the figure accordingly.**

Line 232 : add a reference to Jung et al., in press, as in line 598, for the identification of the origin of replication

**We have now included the reference, as suggested by the reviewer.**

Lines 244-249 : I completely disagree with the choice made to include differences with p-values <0.25 and log2-fold difference of >0.5. Moreover, this is not crucial to deliver the main message behind this analysis. I propose to remove this part of the analysis

**In our opinion, it is one of the main findings of our study that the regulatory responses mediated by the downstream part of the CtrA regulatory network (ChpT and CckA) are considerably more pronounced than those mediated by the upstream part (DivJ, PleC and DivK). To present this result in a meaningful and clear way, it is important to compare how many genes are regulated by each of these modules and to determine the overlap in the number of commonly regulated genes and gene functions. Therefore, we feel that it is crucial to define meaningful regulons.**

**Taking into account the small differences in expression levels between the wt and *divJ, pleC* and *divK* mutants, we felt that using a log2-fold change of 1.3 as a threshold was too strict. Turning to literature, we found that even though the stricter log2-fold difference threshold of 1.3 is more commonly used, a log2-fold threshold of 0.5 has been reported in numerous papers (e.g. Manry et al., 2017, PLoS Genet). In fact, the study that defined the DivJ and PleC regulons of *C. crescentus* used a log2-fold change of 0.58 to identify genes that were differentially regulated in the corresponding mutants (Chen et al., 2006). However, when analyzing small expression differences, even small variations between the triplicates lead to relatively high p-values. There­fore, a higher p-value cut-off is needed. In older microarray studies (e.g. many performed on *C. crescentus*), p-values are often not considered at all. We thus feel that, in light of relevant literature, our thresholds are reasonable.**

**We agree with the reviewer that due to this choice of thresholds some false positives might be included in the DivJ, PleC and DivK regulons that we define. However, since these regulons contain many genes that either are cell-cycle related or represent functions present in the respective regulons of *C. crescentus*, we are convinced that most of the genes that we include in the regulons are in fact differentially regulated in the respective mutants. However, we have now included a short discussion of this issue in the manuscript. Please see L265-266 of the revised manuscript.**

Line 261 : the data reported here clearly show that the the upstream and downstream parts of the DivK-CtrA network are clearly uncoupled for a large fraction of target genes, but it does not shown that "only part of the signal is transmitted to the next protein at EACH step" since CckA and ChpT-controlled regulons are very correlated

**Adjusted according to the comments by the reviewer (see L282 of the revised manuscript).**

Line 263 : I would not argue that CtrA is not controlled at the level of proteolysis because it could be controlled in other conditions, not tested here. I suggest to mention that the half-life of CtrA in C. crescentus and H. neptunium are very different, at least along the cell cycle in the conditions tested here.

**We have now made more explicit that the statements only apply to the conditions that we tested. Please see L297 of the revised manuscript.**

Line 289 : Figure 11E,F are the data in Fugure 11F really extracted from the blots shown in Fig. 11E? In Figure 11E it seems that CtrA level is more strongly decreased in the ∆rcdA strain compared to WT and ∆cpdR, but maybe isn't it reproducible?

**Indeed, the data shown in Fig. 11F are based on the data in Fig. 11E (plus two additional replicates per strain). However, the data do not directly represent the intensity values obtained from the Western blots, but they were corrected for additional growth that took place after the addition of chloramphenicol. The Western blots shown in Fig. 11E were made using samples that were normalized to the same OD580. However, if cells continue to divide after the addition of chloramphenicol, CtrA is further diluted by its distribution to the daughter cells. To account for this effect, we monitored the growth of these strains after addition of chloramphenicol and corrected the values extracted from the blots, so that in the end we obtained the amount of CtrA in the culture, which is the relevant value. We had described this in L669-672 of the original manuscript. We have now also clarified this in the figure legends.**

Lines 289-290 : why not adding "as in C. crescentus" after "CtrA is not regulated"?

**We have changed the manuscript accordingly (see L311 of the revised manuscript).**

From line 344 : I would remove the Discussion about the flow of signal and its quantification. This is the only really weak part of the manuscript and it does not contribute to the main conclusion of the paper. Such considerations should also be removed from the abstract. I would also remove Figure 12.

**In our opinion, this analysis is a relevant novel way of looking at signaling pathways, and more specifically, at the CtrA network. In our manuscript, this analysis can help explain, for instance, the finding that in *H. neptunium* DivK can be knocked out without any obvious phenotype, which is one of the major differences between the CtrA pathway in *H. neptunium* as compared to *C. crescentus*. As we mention in L364-371 (revised manuscript), such analyses could shine light on so-far ununderstood aspects of the CtrA pathway in other organisms as well. The analysis will also enable more quantitative comparisons between species in the future. As we believe that this part of the manuscript makes an important point and reviewer 3 sees it as a major strength of our work, we would like to keep this discussion in the manuscript**

Lines 629-634 : I would remove this part about quantification of "signal" (see above).

**We would prefer to keep this analysis in our manuscript (see above).**

**Reviewer #2:**

Hyphomonas neptunium is an emerging model to study the diversity of pathways regulating the bacterial cell cycle and the Thanbichler lab pioneers to reach this new goal. In this study, they made an extremely comprehensive analysis of the impact of the CtrA response regulator and of its post-transcriptional regulators (kinases, proteases…) on the regulation of the quite original cell cycle of this bacterium (it divides through buds that form at the tip of a stalk-like structure) and made fruitful comparisons with the well-known Caulobacter model. They constructed a long series of deletion or depletion mutants for each of the regulators upstream of CtrA and perfomed RNA-Seq analysis, which were combined with ChIP-Seq experiments using CtrA antibodies, to define the direct CtrA regulon in this bacterium. In addition, they engineered strains encoding fluorescently-labeled regulators to look at their subcellular localization as a function of the cell cycle and used BTH and in vitro phosphorylation assays, to reveals how and where key regulators are phosphorylated and active in cells. Altogether, their results demonstrate that the network of genes regulated by CtrA diverged significantly between the two compared bacteria, despite the fact that they are close relatives at the genome level. They also show that the regulatory network controlling CtrA activity and CtrA levels mostly includes the same actors as in Caulobacter, while their function has, again, significantly evolved, illustrating extraordinary plasticity. Their model finally suggests the existence of novel regulators that still need to be identified in this emerging model, providing interesting perspectives. The only things that are regrettable, is that they could not obtain a CtrA-depletion strain due to technical constrains (that are fully understandable) and that experiments addressing the impact of CtrA on replication control (through binding to the chromsosomal origin) in H. neptunium remain limited in this study (see “Major comment” below). Overall, the manuscript is however very informative and carefully written.

Major comments:

1) Lines 145-147: Authors could test if mutants over-replicate their chromosome much more directly through an estimation of the ori/ter ratio using Q-PCR. This would be informative, since one of the major functions of CtrA, besides its activity as a transcription factor, is to control replication initiation.

**We agree that FACS analysis can only provide circumstantial evidence for a, direct or indirect, role of CtrA in replication initiation (as also suggested by the fact that CtrA was found by ChIP to bind close to the replication origin). However, since the mutant cells have a severe cell cycle and cell division defect, knowledge about the *ori/ter* ratio would still not allow us to draw any firm conclusions on the involvement of CtrA in replication control. Any effect observed could be indirect, caused by the misregulation of replication factors that are part of the CtrA regulon.Apart from that, experience shows that with qPCR it is difficult to clearly show an elevated ori/ter ratio unless the ratio is very high.**

2) I note that many promoters that are supposedly bound to CtrA in vivo (from ChIP-Seq data in Fig.8F) are not significantly mis-regulated in cckA and chpT mutants (from RNA-Seq = genes described as “Entire regulon” in Fig.8F). Could this mean that certain genes (up to 284 of them) are regulated by CtrA independently of the phosphorylation status of CtrA? Did they still find many promoters with a CtrA binding motif in this set of genes? It may be something interesting to discuss.

**We agree with the reviewer that this finding deserves more attention. We have therefore extended our discussion (see L225-229; in addition to the discussion that was already present in L222-225). We indeed identified CtrA-binding motifs upstream of these 284 genes. As suggested by the reviewer, the fact that many CtrA-bound genes are not misregulated after CckA and ChpT depletion could indeed be explained by phosphorylation-independent CtrA binding (now mentioned in L223). However, there a two additional explanations: first, there may be genes whose expression is upregulated by CtrA in one part of the cell cycle and downregulated by CtrA in another part of the cell cycle, so that they on average appear non-regulated in RNA-seq analyses of mixed cultures (as performed in our study). In this case, the inactivation of CtrA would not have any effect on the apparent (average) expression levels. Second, residual phosphorylation of CtrA by the basal levels of CckA and ChpT left after depletion may be sufficient to mediate the binding of CtrA to some high-affinity promoters.**

**Notably, in *C. crescentus*, there are also a number of CtrA-bound genes whose expression levels remain unchanged in a CtrA loss-of-function mutant (Laub et al, 2002). Our findings may thus reflect a general feature of the CtrA regulatory pathway in alphaproteobacteria.**

Minor comments:
1) Author Summary (lines 18-19): Maybe you should also mention that CtrA is not only a transcriptional regulator, but also a repressor of replication initiation that binds to the chromosomal origin.

**We have included this information in the author summary. Please see L23-24 of the revised manuscript.**

2) Although Fig.1A is essentially a review of the CtrA regulatory network in Caulobacter, I think it is important to keep it in, considering the complexity of this network.

**We are happy that the reviewer shares our concern that the reader may have difficulties to understand the paper without a figure summarizing the current knowledge of the CtrA pathway in *C. crescentus*.**

3) Line 43: replace “offspring” by “offsprings”

**We prefer “offspring" as the plural form over “offsprings”.**

4) Lines 172-174: can you please add a few references there

**We have now included multiple relevant references to this sentence.**

5) Line 270: change “course the cell cycle” for “course of the cell cycle”

**Done. Thank you for pointing out this mistake.**

6) Line 272: delete “at”

**Done.**

7) Line 317: change for “The observations that DivK is dispensible and that its lack has no obvious…”

**Changed.**

8) Line 404: “C. crescentus” (typo)

**Corrected.**

9) Line 507: delete “a”

**Done.**

10) Line 1400: add “.” in front of “The”

**Done.**

**Reviewer #3:**

This manuscript addresses systems-level connections among an important and intensely studied group of regulatory proteins in H Neptunium, a species that serves as a comparator with the related and far better understood model species C. crescentus. It is an unusually broad and far-reaching study, and assembles a large amount of experimental data around a model that provides some interesting insight into a complex signaling pathway. A cynic would say that the main conclusion in comparing these two related species is that they are mostly the same but a bit different, and that this is not a very interesting result. However, the manuscript strives to go steps beyond this and describe how the details of pathway connectivity can change while its overall structure is maintained. Understanding such evolutionary turning points are important, particularly in light of other studies in more distantly related bacteria, which show that the CtrA pathway is no longer essential and reduced to a more limited role in the cell cycle. Clearly the pathway has undergone a substantial degree of evolutionary change, but we don’t understand how this is possible for a core pathway that is essential for life in some species.

The manuscript is mostly successful in striving to describe the wiring of this similar-but-different system, but it is missing two key points. One is positive evidence that the phosphorylation state of H. Neptunium CtrA has an effect on cell division / cell physiology, and the other is better points of comparison with C. crescentus vis a vis the leaky pipeline model. These points are described in greater detail in the major comments section below.

MAJOR COMMENTS

Figure S8, Figure 11, Figure 12: The manuscript shows that H. Neptunium CtrA is an essential gene and provides further evidence supporting the idea that it acts as a master regulator of gene expression and chromosome replication, as it does in Caulobacter. The manuscript also shows that H Neptunium CtrA is not degraded by CpdR/RcdA mediated proteolysis, and that CtrA protein levels are stable over the cell cycle. Having ruled out proteolysis as a regulatory mechanism, the other likely mode of regulation for CtrA is phosphorylation. This type of mechanism is very strongly implied by the author’s phosphotransfer signaling model in Figure 12, the in vitro phosphotranfer experiments in earlier figures, and in many other places throughout the manuscript text. But a major gap in the data is that CtrA phosphorylation states are never explored experimentally. Several potential ways of solidifying this important part of the manuscript’s conclusions are suggested as options below:
+ In Figure S8, it would be helpful to determine whether CtrA is differentially phosporylated relative to protein level under CckA and ChpT depletion conditions. Residual phosphorylation might also provide some evidence for parts of the “leaky pathway” model in Figure 12.
+ In Figure 11E, it might be informative to determine whether the half-life of phospho-CtrA in H. Neptunium is less than the half-life of the protein. This would suggest that the phosphorylation level of the protein is changed during its lifetime, strongly implying regulation by phosphosignaling.
+ Determining the fate of Phospho-CtrA in synchronized cultures and comparing that signal to CtrA protein levels (as shown in Figure 11A) may help to demonstrate phospho-regulation.
+ It might be possible to see phenotypes with the expression of a mutant form of CtrA, for example the phosphomimetic D51E and de-phospho D51A variants. Perhaps one of these will have a strong phenotype (cell shape, chromosome number, etc…) relative to wildtype CtrA, which would provide some evidence for the phosphoregulation model.

**We agree that direct evidence for *in vivo* CtrA phosphorylation would provide additional support to our findings. We have therefore tried intensively to adapt phos-tag gels to the detection of phosphorylated CtrA in *H. neptunium* cell lysates. We did occasionally see a faint, slightly slower-migrating band for the wild-type strain. However, it was not possible to obtain consistent, reproducible data, suggesting that the phosphorylated state of CtrA may be very unstable. In line with the notorious instability of aspartophosphate residues, it was also not possible to detect phosphorylated CtrA by mass spectrometry.**

**We also tried to affect cell cycle regulation by producing the CtrA-D51E variant in the *H. nep­tu­nium* wild-type background. However, since there are no replicating plasmids available for *H. neptunium*, this could only be achieved by integrating a single copy of the corresponding allele downstream of the chromosomal Pcop promoter. Induction of the promoter did not have any obvious effect on cell morphology or growth. This result was to be expected, because in *C. crescentus*, the CtrA-D51E variant only had an adverse effect when highly overexpressed from a high-copy plasmid and stabilized by fusion to a C-terminal FLAG tag that prevented its ClpXP-dependent degradation. However, the *H. neptunium* Pcop promoter is relatively weak and not suitable for protein overexpression. In fact, its activity is not even close to that of the *ctrA* promoter, which prevented us from constructing a conditional mutant expressing *ctrA* under Pcop control. Therefore, we find it difficult to draw any conclusions from experiments with inducible CtrA variants. At this point, we therefore have to conclude that the limited toolset available for *H. neptunium* prevents us from verifying the *in vivo* phosphorylation state of CtrA.**

**However, the CckA-ChpT-CtrA pathway is highly conserved in the alphaproteobacteria and we show phosphotransfer between ChpT and CtrA under stringent conditions *in vitro*. Furthermore, a significant number of genes in the CckA and ChpT regulons show a CtrA consensus binding motif in their promoter region. These data strongly suggest that CtrA is the target of the CckA-ChpT pathway.**

Figure 8F: Is it unusual to find that only 1/3 of the genes that have a CtrA binding site nearby are part of the “direct CtrA regulon” under CckA and ChpT? Is this further evidence for the leaky pipeline model? It would be helpful to know how this compares with the Caulobacter CtrA regulon (as defined by the genes under direct control of CckA / ChpT) and the array of CtrA binding sites in the Caulobacter genome.

**We were also surprised to find so many genes that have a CtrA binding site that were not found to be significantly up- or downregulated in the CckA and ChpT depletion backgrounds. As pointed out above in our response to reviewer 2, this observation may be explained by two effects: first, there may be genes whose expression is upregulated by CtrA in one part of the cell cycle and downregulated by CtrA in another part of the cell cycle, so that they on average appear non-regulated in RNA-seq analyses of mixed cultures (as performed in our study). In this case, the inactivation of CtrA would not have any effect on the apparent (average) expression levels (see L225-227 of the revised manuscript). Second, residual phosphorylation of CtrA by the basal levels of CckA and ChpT left after depletion may be sufficient to mediate the binding of CtrA to some high-affinity promoters (see L222-225 of the revised manuscript). However, it is also conceivable that some of the genes, whose expression was affected by CckA and/or ChpT depletion, are not regulated by CtrA at all.**

**We agree with the reviewer that it would be very interesting to compare the number of genes with a CtrA binding site that are not affected by CckA and/or ChpT depletion between *H. neptunium* and *C. crescentus*. We have now performed this analysis on published *C. crescentus* microarray data for the CckAts (Jacobs et al, 2003) and ChpT depletion (Biondi et al., 2006) strains using the CtrA binding sites reported by Laub et al (2002). These papers do not provide p-values for the expression levels, which forced us to consider exclusively the log2-fold differences in the expression levels. The percentage of CtrA-bound sites that are part of the CckA and/or ChpT regulon was only 62% when using a log2-fold difference of 0.81 as a threshold (as reported for the CtrA microarray data by Laub et al, 2002) and 41% when using a log2-fold difference of 1.3 (as done in our study). The reviewer’s comment made us have a close look at the literature, and we found that even when the CtrA regulon was defined directly by using a lack of function mutant of CtrA, still only part (76%) of the CtrA-bound genes were found to be differentially expressed upon CtrA depletion (see Laub et al., 2002). We have chosen not to show this analysis of the *C. crescentus* data in the paper, because the lack of p-values prevents a thorough, statistically valid comparison. But we do now point to the findings of Laub et al (2002) in our revised manuscript (see L227-229).**

L260: The meaning of “part of the signal is transmitted to the next protein at each step” isn’t clear and could have multiple interpretations. Do you mean that these proteins have multiple downstream effectors? Or are there multiple upstream signals? This statement should be restated more clearly. As with the previous comment, this interpretation would be strengthened by including a comparison with Caulobacter (if the data is present in the literature). What percentage of genes in Caulobacter DivJ / PleC / DivK regulons are shared with the Caulobacter CtrA regulon? More broadly, does the leaky pipeline model presented in this manuscript claim that the pathway is more leaky in H. Neptunium than in Caulobacter? If so, what is the evidence for this?

**We have now clarified this statement (see L288-291 of the revised manuscript). What we aim to say is that part of the signal leaves the CtrA regulatory network and that the misregulation of certain genes is not caused by CtrA but by other transcription factors. This signal loss appears at many nodes. For instance, a large part of the genes misregulated in a Δ*divJ* mutant are not misregulated in a Δ*divK* mutant, suggesting that these genes are not regulated through DivK but through another response regulator. Apart from that, as many genes regulated by CtrA are not part of the DivJ and PleC operons, we also expect additional histidine kinases that act as input, but this is not what the sentence pointed out by the reviewer refers to.**

**We agree that a comparison with the situation in *C. crescentus* would be very interesting. We surveyed the literature for gene expression data comparing the *C. crescentus* wild type to relevant mutants. Unfortunately, in some cases, part of the data and details on the analysis pipelines are missing, because they were included in supplements that are no longer available online and communication with the authors did not clarify the issues. Ultimately, we used the expression values available for *divJ* and *pleC* mutants as well as for CckA and ChpT depletion strains. We used the log2-fold differences in expression reported in the respective papers to determine if the genes present in the CtrA direct regulon (as determined by Laub et al., 2002), are regulated by these proteins as well. Although our analysis has a rather preliminary character, since we compare datasets generated in part by different labs, it is now added as File S14 and mentioned in L372-374 and L376-377 of the revised manuscript. In general, DivJ and PleC appear to provide only part of the input signal (suggesting the presence of additional histidine kinases as part of the CtrA network). Moreover, not all genes present in the CtrA regulon are part of the CckA and ChpT regulons, suggesting that either part of the signal bypasses these factors, or part of the signal enters the known CtrA network after these nodes.**

The abstract says that the “essential core pathway is reduced”, which probably refers to the finding that DivK is essential in C. crescentus but non-essential in H Neptunium. But the model in Figure 12 includes another protein, “Box B”, which may work in parallel with DivK, and may itself be essential or synthetic lethal with DivK. If either of these scenarios for Box B turn out to be true, it does not seem correct to say that the essential core pathway is reduced compared to Caulobacter.

**The reviewer is correct. In fact, the essential core pathway may even turn out to be larger in comparison to other organisms once the factor(s) acting in parallel to DivK have been identified. We have now rephrased the text accordingly and also mentioned the protein names in the abstract to clarify what this statement refers to.**

Figure 12: The methods and interpretations related to this figure are interesting. Are there any other publications that have used this kind of data to generate something that looks like a pathway flux analysis or is this a novel method?

**We are not aware of any publication that reports a quantitative comparison of the regulons in a signaling pathway and therefore devised this approach ourselves. We have now clarified this in the manuscript (see L108 and L356-357 of the revised manuscript).**

Figure 3C, S5: the use of the term “aberrant” is vague. In the methods section, provide more explanation of what qualifies as aberrant. How long does a stalk need to be in order to be considered aberrant? How does the calculation work for cell body shape?

**We have now explained the criteria that were used in our manual analysis in more detail in the methods section. See L491-494 of the revised manuscript.**

MINOR COMMENTS

Figure 8 B,E, G: The pie chart diagrams and most of the information they contain are not particularly helpful. I suggest moving them to supplementary data.

**We have now removed the pie charts from Fig. 8 and created a new supplementary figure that displays these pie charts (see Fig. S9 of the revised manuscript).**

L219-221: The manuscript shows a correlation between the presence of CtrA biding sites and changes in expression for these particular genes, but the connection has not been directly demonstrated. This is suggestive, but does not amount to proof. Please adjust the tone of this statement accordingly. Also, it seems the data these lines are referring is shown more effectively in 8H, not 8G.

**We have adjusted the statement accordingly (see L237-239 of the revised manuscript). Please keep in mind that the panels of this figure have been renamed.**

L30: Please change “this right timing” to “the correct timing”.

**Done.**

L36: It is a bit confusing to think of it as “just a kinase and response regulator”, since the system is tied to input signals and output responses. Maybe “…or two components systems (TCSs) when the context is limited to signaling kinases and downstream response regulators.”

**Done. Thank you for this suggestion.**

Figure S9: Please change “considerable” in title to “considerably”.

**Done. Thank you for pointing out this mistake.**

Figure 11D: Please attach “WB” and “LC” labels to the images, as done in 11E.

**Done.**

L235: “does not only provide insight…” Please re-state more clearly.

**Done. Please see L254-256 of the revised manuscript.**

Figure 12: Text sections in figure legends and main text refer to rectangles or boxes, but the shapes are more like ovals. Perhaps “component A,B, C…” is better?

**We have changed the manuscript accordingly.**

Figure 8F: Why is it 284 genes instead if the 286 genes identified in 8E?

**Thank you for pointing out this mistake. We have now corrected this number throughout the manuscript.**

Reference #74 is from BioRxiv. Is this permissible?

**In the meantime, this work has been published, so we have changed the reference accordingly.**

The meaning of the asterisk in Figure 12 is not explained in the figure legend.

**We have added this information to the figure legend.**

File S9: CckA towards ChpT is 57% in field F60 and 60% in C32.

**Thanks for pointing out this mistake! We have now changed the value in field F60 to 60% (as this was the correct value).**
