# Supplementary material for: Integrative and quantitative view of the CtrA regulatory network in a stalked budding bacterium
Source: PLoS Genet. 2020 Apr 23;16(4):e1008724. doi: 10.1371/journal.pgen.1008724 (PMC7200025; doi:10.1371/journal.pgen.1008724)
Supplement: Attachment — Submitted filename: Response to reviewers II.docx [file pgen.1008724.s033.docx]

We thank all reviewers for going though the revised version of our manuscript and appreciate the efforts of reviewer #3 to further improve the paper. Please see below for our responses to the remaining issues.

Reviewer #3:

Abstract: I still have problems with “essential core of the pathway is reduced….” statement in the abstract, for the same reasons that I stated in my first round of comments. I think it is better to avoid “reduced” and use other language. The essential elements of the pathway differ?

We agree that the wording we used was not correct. We have now changed the abstract and pointed out the differences to the *Caulobacter* system more clearly.

The stated reasoning for why 2/3 of genes in CtrA regulon might not vary in a CckA/ChpT depletion is tenable, but weak. I can imagine tight-binding of CtrA-P outlasting depletion or see-sawing effects in different cell types explaining the observed result for some of the genes with CtrA binding sites, but not 2/3 of them. The idea that there is differential binding of CtrA versus Phospho CtrA is plausible but I’m not sure if its supported by evidence from the literature. Generally speaking, doesn’t this result provide further support for a braided signaling network like the one shown in Figure 12?

This is definitely true. We have now included this point in the Discussion.

Unfortunately we still don’t have proof that CtrA is phosphorylated in vivo or if those phosphorylation levels are relevant to H. neptunium physiology, and this is a weakness of the manuscript. This discussion point is also relevant in relation to reviewer #2’s note about the possibility that many (or indeed most?) of the genes in the CtrA regulon may be regulated by CtrA independent of its phosphorylation state. Overall, however, the in vitro phosphotransfer data and consistency in genetic phenotypes indicate that phospohtransfer through this pathway in vivo is very likely, and this is enough to support the manuscript’s final models.

Thank you for accepting the present data as evidence that phosphorylation through the CckA-ChpT pathway plays a role in the regulation of CtrA activity.

I agree with the authors’ feeling that Figure 12 is important, even though it is a frustratingly complicated part of the manuscript and it includes incomplete and high p-value type information. For me, the interconnected “braids” of genetic circuitry in a core cell cycle pathway is a major take-home message of the work, and it is important for understanding the large non-overlap of some of the Venn diagrams presented earlier in the work. The effort to relate this analysis to Caulobacter literature is appreciated.

Thank you for sharing the view that this analysis is an important part of the paper.
